# Genetic investigation of 211 Chinese families expands the mutational and phenotypical spectra of hereditary retinopathy genes through targeted sequencing technology

**DOI:** 10.1186/s12920-021-00935-w

**Published:** 2021-03-29

**Authors:** Zhouxian Bai, Yanchuan Xie, Lina Liu, Jingzhi Shao, Yuying Liu, Xiangdong Kong

**Affiliations:** 1 The Genetics and Prenatal Diagnosis Center, The Department of Obstetrics and Gynecology, The First Affiliated Hospital of Zhengzhou University, Zhengzhou, 450052 Henan China; 2 The Department of Ophthalmology, The First Affiliated Hospital of Zhengzhou University, Zhengzhou, 450052 Henan China; 3 The Physical Examination Center, The First Affiliated Hospital of Zhengzhou University, Zhengzhou, 450052 Henan China; 4 The Department of Central Laboratory, The First Affiliated Hospital of Henan University of Science and Technology, Luoyang, 471003 Henan China

**Keywords:** Hereditary retinopathy, Novel mutations, Targeted sequencing, Genetic testing

## Abstract

**Background:**

Hereditary retinopathy is a significant cause of blindness worldwide. Despite the discovery of many mutations in various retinopathies, a large number of patients remain genetically undiagnosed. Targeted next-generation sequencing of the human genome is a suitable approach for the molecular diagnosis of retinopathy.

**Methods:**

We describe a cohort of 211 families from central China with various forms of retinopathy; 95 patients were investigated using multigene panel sequencing, and the other 116 with suspected Leber hereditary optic neuropathy (LHON) were tested by Sanger sequencing. The detected variation of targeted sequencing was verified by PCR-based Sanger sequencing. We performed a comprehensive analysis of the cases using sequencing data and ophthalmologic examination information.

**Results:**

Potential causal mutations were identified in the majority of families with retinopathy (57.9% of 95 families) and suspected LHON (21.6% of 116 families). There were 68 variants of a certain significance distributed in 31 known disease-causing genes in the 95 families; 37 of the variants are novel and have not been reported to be related to hereditary retinopathy. The NGS panel solution provided a 45.3% potential diagnostic rate for retinopathy families, with candidate gene mutations of undefined pathogenicity revealed in another 12.6%of the families.

**Conclusion:**

Our study uncovered novel mutations and phenotypic aspects of retinopathy and demonstrated the genetic and clinical heterogeneity of related conditions. The findings show the detection rate of pathogenic variants in patients with hereditary retinopathy in central China as well as the diversity and gene distribution of these variants. The significance of molecular genetic testing for patients with hereditary retinopathy is also highlighted.

**Supplementary Information:**

The online version contains supplementary material available at 10.1186/s12920-021-00935-w.

## Background

Hereditary retinopathy is one category of the most common genetic retinal diseases causing blindness [1]. Hereditary retinopathy is characterized by heterogeneity of genetic variation and clinical manifestations. The main inheritance patterns include autosomal dominant, autosomal recessive inheritance and X-linked inheritance [2]. Hereditary retinopathy mainly includes retinitis pigmentosa, macular degeneration, Leber hereditary optic neuropathy (LHON) and retinal dysplasia. Retinitis pigmentosa (RP) comprises a group of blinding retinal diseases caused by abnormalities in photoreceptors [3], with main clinical features of progressive visual field defects, night blindness, bone spicules such as retinal pigmentation and abnormal electroretinograms [4]. LHON is a mitochondrial hereditary eye disease that involves retinal ganglion cells, and it eventually results in degeneration and atrophy of the optic nerve [5]. With the popularization and clinical application of gene sequencing technology, an increasing number of disease-causing genes and mutations have been discovered; these genes are mainly expressed in photoreceptor cells and retinal pigment epithelial cells [6]. Overall, a good understanding of retinopathy genes not only provides a theoretical basis for diagnosis and genetic counselling but also supports guidance for gene therapy [7–9].

The study of the genetics of retinopathy is important to enhance our understanding of the molecular aspects of eye development, disease and treatment. In this research, we chose a family-based strategy to determine the exact inheritance pattern and recurrence risk in offspring. Using such a family-based strategy, we can also determine whether phenotype and genotype co-segregate in a family, which helps to estimate the pathogenicity of candidate mutations. More than half of the patients in this study were suspected of having LHON; direct Sanger sequencing of mitochondrial DNA was performed for some, and next-generation sequencing (NGS) was carried out for the remainder. Despite the discovery of pathogenic mutations and genes of various types of retinopathy, many unknowns remain. Our study will increase knowledge of the mutations and phenotypes of diseases and provide more population information on pathogenic variants. Our research will also illustrate the importance of targeted NGS in the aetiological detection of hereditary eye diseases.

## Methods

### Subject recruitment

In total, 211 Chinese families with retinopathy from central China were recruited for this study, including 116 patients from different families with suspected monocular or binocular LHON and 95 families with other retinopathies. The inclusion criteria for LHON included (1) optic neuropathy and (2) a rapid decline in visual acuity for unknown reasons. The inclusion criteria for other retinopathies included (1) retinitis pigmentosa; (2) macular degeneration (MD); and (3) multiple fundus lesions or retinal dysplasia. Of the patients, 116 (Part A) with fundus optic atrophy were subjected to LHON Sanger sequencing, and targeted NGS for other retinopathies was performed for 95 (Part B); the patients were examined and diagnosed by the Ophthalmology Department. The specific clinical manifestations of the patients were recorded. Samples were obtained with written informed consent. The retinopathy patients sought medical and genetic consultations in the hospital during 2017 and 2019, and 4 mL peripheral blood was from individuals in 211 retinopathy families. The clinical data for the patients were collected at the outpatient clinic.

The subjects of Part A underwent Sanger sequencing that included specific pathogenic sites of mitochondrial DNA; the mother of a subject carrying pathogenic mitochondrial variants underwent the same test to explore the source of the variation. The proband of the families of Part B was screened by targeted NGS. Then, the parents of the proband and other members of the family were tested by Sanger sequencing to detect and verify the carrying status of candidate mutations screened through targeted NGS.

### Targeted next-generation sequencing and sanger sequencing

Genomic DNA was extracted from EDTA-treated blood samples using a Blood DNA Midi Kit D3494 (Omega Bio-tek, USA) with nucleic acid automatic extraction equipment (Eppendorf epMotion 5075 m, Germany). A customized panel (MyGenostics Inc., China) capturing 463 known genes (Additional file 1: Table S1) related to retinal disease was designed to detect the genetic cause of the congenital retinopathy in the families. Panel sequencing was conducted using the Illumina NextSeq500 system in our clinical lab. The average sequencing depth of the target panel sequence was more than 100 × , and the coverage was 98%. Version GRCh37 is the human reference genome used for short-read mapping (https://www.gencodegenes.org/human/release_37lift37.html). The transcript RefSeq number was obtained from the Ensembl database (http://asia.ensembl.org) (Tables 1 and 2) [10]. PCR-based Sanger sequencing was used to validate disease-causing mutations based on NGS. The carrying status of a novel mutation in other family members was also assessed by Sanger sequencing. The primers used for PCR were designed by GeneTool software. A capillary electrophoresis apparatus (ABI 3130xl, USA) and dGTP BigDye® Terminator sequencing kit (ABI, USA) were used for Sanger sequencing.

**Table 1:** General situation of families with pathogenic or likely pathogenic mutations

Fa	Np	Gene	Transcript RefSeq	Ex	NA Changes	AA changes	Hzyo	Pf	Reported	Gm	Disease	SPM	ACMG grade
14	2	*RHO*	NM_000539	1	c.251 T > C	p.L84P	Het	–	Novel	AD	RP, 4	+ + +	PS4 + PM1 + PM2 + PP1 + PP3
15	4	*RHO*	NM_000539	2	c.403C > T	p.R135W	Het	0/ 1.082^e−5^	Yes[36]	AD	RP, 4	+ + +	PS1 + PM1 + PP1 + PP3
48	1	*RHO*	NM_000539	3	c.541G > A	p.E181K	Het	–	Yes[37]	AD	RP, 4	+ + +	PS2 + PM2 + PP3
54	2	*RHO*	NM_000539	2	c.403C > T	p.R135W	Het	0/ 1.082^e−5^	Yes[38]	AD	RP, 4	+ + +	PS1 + PM1 + PP1 + PP3
18	3	*NDP*	NM_000266	2	c.124C > A	p.H42N	Hemi	–	Novel	XL	FEVR2[39]	+ + +	PM2 + PM5 + PP1 + PP3
32	1	*NDP*	NM_000266	3	c.343C > T	p.R115X	Hemi	–	Yes[40]	XLR	Norrie	+ + +	PVS1 + PS1 + PM2 + PP3
46	1	*NDP*	NM_000266	3	c.401_402delGA	p.*134Wfs*13	Hemi	–	Novel	XL	FEVR2	/ / /	PVS1 + PS2 + PM2
55	3	*NDP*	NM_000266	3	c.268C > T	p.R90C	Hemi	–	Yes[41]	XLR	Norrie	+ + +	PS1 + PM2 + PP1 + PP3
7	1	*USH2A*	NM_206933	2	c.99_100insT	p.R34Sfs*41	Hom	6.242 ^e−5^/ 3.231^e−5^	Yes[42]	AR	Usher 2A	/ / /	PVS1 + PS1 + PM2
9	1	*USH2A*	NM_206933	55	c.10859 T > C	p.I3620T	Het	1.16^e−4^/ 1.219^e−5^	Yes[43]	AR	Usher 2A/RP, 39	+ + +	PS1 + PM2 + PM3 + PP3
		*USH2A*	NM_206933	13	c.2802 T > G	p.C934W	Het	2.441^e−3^/1.915^e−4^	Yes[44]	AR	Usher 2A/RP, 39	+ + +	PS1 + PM2 + PM3 + PP3
47	1	*USH2A*	NM_206933	63	c.13596dupC	p.S4533Lfs*28	Het	–	Novel	AR	Usher 2A	/ / /	PVS1 + PM2 + PM3
		*USH2A*	NM_206933	56	c.10962C > A	p.Y3654X	Het	–	Novel	AR	Usher 2A	+ + +	PVS1 + PM2 + PM3 + PP3
27	1	*RS1*	NM_000330	6	c.598C > T	p.R200C	Hemi	–	Yes[45]	XLR	Retinoschisis	+ + +	PS1 + PM2 + PP3
38	1	*RS1*	NM_000330	4	c.214G > A	p.E72K	Hemi	0/ 1.678^e−5^	Yes[45]	XLR	Retinoschisis	+ + +	PS1 + PM2 + PP3
51	2	*RS1*	NM_000330	4	c.206_207delTG	p.Leu69Argfs*16	Hemi	–	Yes[46]	XLR	Retinoschisis	/ / /	PVS1 + PS1 + PM2 + PP1
1	1	*MERTK*	NM_006343	8	c.1186G > T	p.E396X	Het	–	Yes[47]	AR	RP,38	/ / +	PVS1 + PS1 + PM2
		*MERTK*	NM_006343	3	c.518A > G	p.Y173C	Het	0/ 1.219^e−5^	Novel	AR	RP,38	+ + +	PM2 + PM3 + PP3 + PP4
2	2	*MERTK*	NM_006343	4	c.754delC	p.P252Qfs*3	Hom	–	Novel	AR	RP,38	/ / /	PVS1 + PM2 + PP1

Fa	Np	Gene	Transcript ErfSeq	Ex	NA Changes	AA changes	Hzyo	Pf	Reported	Gm	Disease	SPM	ACMG grade
3	1	*CYP4V2*	NM_207352	7	c.(802–8)_810delTCATACAGGTCATCGCTinsGC	?p.268_270del/ Splicing	Hom	7.963^e−4^/ 6.856^e−5^	Yes[48]	AR	Bietti CCD	/ / /	PVS1 + PS1 + PM2
4	2	*CYP4V2*	NM_207352	7	c.(802–8)_810delTCATACAGGTCATCGCTinsGC	?p.268_270del/ Splicing	Het	7.963^e−4^/ 6.856^e−5^	Yes[48]	AR	Bietti CCD	/ / /	PVS1 + PS1 + PM2 + PM3 + PP1
		*CYP4V2*	NM_207352	7	c.958C > T	p.R320X	Het	0/ 4.061^e−6^	Yes[49]	AR	Bietti CCD	/ / +	PVS1 + PS1 + PM2 + PM3 + PP1
5	4	*FSCN2*	NM_001077182	1	c.72delG	p.T25Qfs*120	Het	0.01238/ 8.801^e−4^	Yes	AD	RP, 30	/ / /	PVS1 + PS1 + PP1
6	2	*FSCN2*	NM_001077182	1	c.72delG	p.T25Qfs*120	Het	0.01238/ 8.801^e−4^	Yes[50]	AD	RP, 30	/ / /	PVS1 + PS1 + PP1
12	4	*PRPF31*	NM_015629	11	c.(1074–8)_1079delGTCCCCAGGTACCG	?p.358_360delRYRinsS/ Splicing	Het	–	Novel	AD	RP, 11	/ / /	PVS1 + PM2 + PP1
50	2	*PRPF31*	NM_015629	12	c.1215delG	p.Val406fs*7	Het	–	Yes[51]	AD	RP, 11	/ / /	PVS1 + PS1 + PM2 + PP1
33	1	*RPGR*	NM_001034853	15	c.2236_2237delGA	p.E746Rfs*23	Hemi	–	Yes	XLR	MD	/ / /	PVS1 + PS1 + PM2
52	2	*RPGR*	NM_001034853	15	c.2236_2237delGA	p.E746Rfs*23	Hemi	–	Yes[52]	XLR	MD	/ / /	PVS1 + PS1 + PM2 + PP1
43	5	*RP2*	NM_006915	3	c.769–2A > G	splicing	Hemi	–	Yes[53]	XL	RP, 2	/ / +	PVS1 + PS1 + PM2 + PP1
44	4	*RP2*	NM_006915	2	c.572_582dup11	p.Pro190Profs*52	Hemi	–	Novel	XL	RP, 2	/ / /	PVS1 + PM2 + PP1
36	1	*ABCA4*	NM_000350	29	c.4352 + 1G > A	splicing	Het	0/ 8.123^e−6^	Yes[54]	AR	Stargardt 1	/ / +	PVS1 + PS1 + PM2 + PM3
		*ABCA4*	NM_000350	13	c.1804C > T	p.R602W	Het	2.904^e−4^/ 4.477^e−5^	Yes[55]	AR	Stargardt 1	+ + +	PS1 + PM2 + PM3 + PP3
11	1	*TULP1*	NM_003322	13	c.1318C > T	p.R440X	Het	0/ 1.145^e−5^	Yes[56]	AR	LCA 15	/ / +	PVS1 + PS1 + PM2
		*TULP1*	NM_003322	12	c.1142 T > G	p.V381G	Het	–	Novel	AR	LCA 15	+ + +	PM2 + PM3 + PP3 + PP4
16	1	*CHM*	NM_000390	5	c.544delT	p.C182Vfs*14	Hemi	–	Novel	XLD	choroideremia	/ / /	PVS1 + PM2
28	1	*RPGRIP1*	NM_020366	16	c.2662C > T	p.R888X	Hom	0/ 1.68^e−5^	Yes[57]	AR	LCA6	+ + +	PVS1 + PS1 + PM2 + PP3

Fa	Np	Gene	Transcript RefSeq	Ex	NA Changes	AA changes	Hzyo	Pf	Reported	Gm	Disease	SPM	ACMG grade
17	2	*PRPF8*	NM_006445	36	c.5792C > T	p.T1931M	Het	–	Novel	AD	RP, 13	+ + +	PM2 + PP1 + PP2 + PP3 + PP4
20	1	*TRPM1*	NM_0012 52,020	21	c.2789 T > A	p.I930N	Het	–	Novel	AR	CSNB1C	+ + +	PM2 + PM3 + PP2 + PP3
		*TRPM1*	NM_0012 52,020	22	c.3178 + 1G > A	splicing	Het	6.889^e−4^/ 5.772^e−5^	Yes[58]	AR	CSNB1C	/ / +	PVS1 + PS1 + PM2
21	1	*NR2E3*	NM_014249	6	c.925C > T	p.R309W	Hom	0/ 8.34^e−6^	Novel	AR	GF	/ + /	PM2 + PM5 + PP2 + PP4
22	1	*PAX2*	NM_003990	2	c.70dupG	p.V26Gfs*28	Het	0/ 1.237^e−5^	Yes[59]	AD	RCS	/ / /	PVS1 + PS1
34	2	*KCNV2*	NM_133497	1	c.506_513delTGCTGCT	p.V169Gfs*40	Het	–	Novel	AR	RCD3B	/ / /	PVS1 + PM2 + PM3 + PP1
		*KCNV2*	NM_133497	1	c.137G > A	p.W46X	Het	–	Yes[60]	AR	RCD3B	+ + +	PVS1 + PS1 + PM2 + PP1 + PP3
35	2	*FZD4*	NM_206933	2	c.612 T > A	p.C204X	Het	–	Novel	AD	FEVR1	+ + +	PVS1 + PM2 + PP1 + PP3
37	2	*LRP5*	NM_002335	2	c.485_488delACGG	p.H162Rfs*38	Het	–	Novel	AD	FEVR4	/ / /	PVS1 + PM2 + PP1
39	1	*SLC38A8*	NM_001080442	7	c.927_928delCT	p.Y310Pfs*57	Het	–	Novel	AR	FH2	/ / /	PVS1 + PM2 + PM3
		*SLC38A8*	NM_001080442	6	c.697G > A	p.E233K	Het	2.778^e−4^/ 6.886^e−5^	Yes[61]	AR	FH2	+ + +	PS1 + PM2 + PP3
40	1	*AIPL1*	NM_001285399	3	c.385C > T	p.Q129X	Hom	–	Novel	AR	LCA4	+ + +	PVS1 + PM2 + PP3
41	1	*FRMD7*	NM_194277	10	c.910C > T	p.R304X	Hemi	0/ 5.608^e−6^	Yes[62]	XLR	Nystagmus 1	+ + +	PVS1 + PS1 + PM2 + PP3
42	1	*GUCY2D*	NM_000180	18	c.3177_3178delAC	p.R1060Rfs*11	Hom	0/ 4.935^e−6^	Novel	AR	LCA4	/ / /	PVS1 + PM2
45	1	*CNGA1*	NM_001142564	5	c.472delC	p.L158Ffs*4	Het	0.0012/ 6.455^e−5^	Novel[63]	AR	RP, 49	/ / /	PVS1 + PM2
		*CNGA1*	NM_001142564	5	c.453C > A	p.Y151X	Het	5.798^e−5^/ 4.068^e−6^	Novel	AR	RP, 49	+ + +	PVS1 + PM2 + PP3
49	3	*TSPAN12*	NM_012338	8	c.731delT	p.L244Rfs*17	Het	0/ 4.064^e−6^	Novel	AD	EV5	/ / /	PVS1 + PM2 + PP1

Fa denotes Family No.; Np denotes the number of patients; Ex denotes an exon; NA denotes nucleic acid; AA denotes amino acid; Hzyo denotes heterozygosity; Pf denotes the population frequency recorded in the gnomAD database; Gm denotes the genetic model; Disease denotes OMIM disease; SPM denotes SIFT, PolyPhen_2 and Mutation t@sting predicting, ‘ + ’denotes damaging, ‘-’denotes benign, and ‘/’ denotes no data. RP,4 denotes retinitis pigmentosa, type 4; FEVR2 denotes familial exudative vitreoretinopathy, type 2; Usher 2A denotes Usher syndrome, type 2A; RP,39 denotes retinitis pigmentosa, type 39; RP,38 denotes retinitis pigmentosa, type 38; Bietti CCD denotes Bietti crystalline corneoretinal dystrophy; RP, 30 denotes retinitis pigmentosa, type 30; RP, 11 denotes retinitis pigmentosa, type 11; MD denotes macular degeneration, X-linked atrophic; RP,2 denotes retinitis pigmentosa, type 2; Stargardt 1 denotes Stargardt's disease, type1; LCA 15 denotes Leber congenital amaurosis, type 15; LCA6 denotes Leber congenital amaurosis, type 6; RP,13 denotes retinitis pigmentosa, type 13; CSNB1C denotes congenital stationary night blindness, type 1C; GF denotes Goldmann-Favre syndrome; RCS denotes renal coloboma syndrome; RCD3B denotes retinal cone dystrophy, type 3B; FEVR1 denotes familial exudative vitreoretinopathy, type 1; FEVR4 denotes familial exudative vitreoretinopathy, type 4; FH2 denotes foveal hypoplasia, type 2; LCA4 denotes Leber congenital amaurosis, type 4; Nystagmus 1 denotes nystagmus, type 1, congenital, X-linked; RP, 49 denotes retinitis pigmentosa, type 49; EV 5 denotes exudative vitreoretinopathy, type 5

**Table 2:** General situation of families with likely pathogenic mutations or related mutations of undetermined significance

Fa	Np	Gene	Transcript RefSeq	Ex	NA Changes	AA changes	Hzyo	Pf	Reported	Gm	OMIM Disease	SPM	ACMG grade
8	1	*USH2A*	NM_206933	41	c.8002G > T	p.E2668X	Het	–	Novel	AR	Usher 2A/RP, 39	/ / +	PVS1 + PM2
		*USH2A*	NM_206933	13	c.2802 T > G	p.C934W	Het	2.441^e−3^/1.915^e−4^	Yes[44]	AR	Usher 2A/RP, 39	+ + +	PS1 + PM2 + PP3
10	1	*USH2A*	NM_206933	63	c.12608A > G	p.Q4203R	Het	9.457^e−3^/ 3.677^e−3^	Novel	AR	Usher 2A/RP, 39	–	PM2 + BP4
		*USH2A*	NM_206933	22	c.4758 + 3A > G	Splicing	Het	1.855^e−2^/ 1.457^e−3^	Yes[64]	AR	Usher 2A/RP, 39	/ / /	PS1 + PM2
23	1	*USH2A*	NM_206933	66	c.14411G > A	p.G4804E	Het	–	Novel	AR	Usher 2A/RP, 39	/ / +	PM2
		*USH2A*	NM_206933	19	c.4217C > A	p.S1406X	Het	–	Novel	AR	Usher 2A/RP, 39	+ + +	PVS1 + PM2 + PP3
53	1	*USH2A*	NM_206933	65	c.14287G > A	p.G4763R	Het	–	Yes[65]	AR	Usher 2A/RP, 39	+ + +	PS1 + PM2 + PP3
		*USH2A*	NM_206933	4	c.784 + 2 T > G	Splicing	Het	–	Novel	AR	Usher 2A/RP, 39	/ / /	PVS1 + PM2
19	1	*USH1C*	NM_153676	5	c.407G > A	p.R136Q	Het	1.16^e−4^/ 1.223^e−4^	Novel	AR	Usher 1C	/ / +	PM2
		*USH1C*	NM_153676	15	c.1250C > T	p.T417I	Het	–	Novel	AR	Usher 1C	/ / +	PM2
13	1	*BBS2*	NM_031885	6	c.626 T > C	p.L209P	Het	–	Yes[66]	AR	RP, 74	+ + +	PS1 + PM2 + PP3
		*BBS2*	NM_031885	1	c.79A > C	p.T27P	Het	–	Novel	AR	RP, 74	–	PM2 + BP4
25	1	*LRP5*	NM_002335	15	c.3361A > G	p.N1121D	Het	7.528^e−3^/ 5.616^e−4^	Yes[67]	AR	FEVR4	+ + +	PS1 + PM2 + PP3
		*LRP5*	NM_002335	18	c.3901G > A	p.A1301T	Het	2.403^e−3^/ 2.149^e−4^	Novel	AR	FEVR4	−	PM2 + BP4
56	1	*LRP5*	NM_002335	15	c.3377 T > C	p.L1126P	Het	–	Novel	AR	FEVR4	+ + +	PM2 + PP3
		*LRP5*	NM_002335	22	c.4519G > T	p.D1507T	Het	–	Novel	AR	FEVR4	+ + +	PM2 + PP3
24	1	*ABCA4*	NM_000350	5	c.553C > T	p.Q185X	Het	–	Yes[68]	AD	AMD2	+ + +	PVS1 + PS1 + PM2 + PP3
26	1	*RS1*	NM_000330	4	c.240G > C	p.Q80H	Hemi	–	Novel	XLR	Retinoschisis	/ / +	PM2 + PP4
29	1	*GPR143*	NM_000273	2	c.263G > A	p.R88Q	Hemi	–	Novel	XL	Nystagmus 6	+ + +	PM2 + PP3
31	1	*FBN2*	NM_001999	30	c.3923dupG	p.C1308Wfs*5	Het	–	Novel	AD	EMD	/ / /	PVS1 + PM2

Fa denotes Family No.; Np denotes the number of patients; Ex denotes an exon; NA denotes nucleic acid; AA denotes amino acid; Hzyo denotes heterozygosity; Pf denotes the population frequency recorded in the gnomAD database; Gm denotes the genetic model; Disease denotes OMIM disease; SPM denotes SIFT, PolyPhen_2 and Mutation t@sting predicting, ‘ + ’ denotes damaging, ‘-’ denotes benign, and ‘/’ denotes no data. Usher 2A denotes Usher syndrome, type 2A; RP,39 denotes retinitis pigmentosa, type 39; Usher 1C denotes Usher syndrome, type 1C; RP,74 denotes retinitis pigmentosa, type 74; FEVR4 denotes familial exudative vitreoretinopathy, type 4; AMD2 denotes age-related macular degeneration, type 2; Nystagmus 6 denotes nystagmus, type 6, congenital, X-linked; EMD denotes macular degeneration, early onset

The patients with LHON were evaluated using PCR-based Sanger sequencing, which included only 3 common mutant sites of mitochondrial DNA, namely, *MTND1*mt.3460, *MTND4*mt.11778 and *MTND6*mt.14484, and 10 rare mutant sites of mitochondrial DNA, namely, *MTND1* (mt.3376, mt.3635, mt.3700, mt.3733), *MTND6* (mt.14482, mt.14495, mt.14502, mt.14568, mt.14498, mt.14325). The pathogenicity of these mitochondrial mutations is known. The PCR primers used were designed with GeneTool software (refer to Additional file 1: Table S2).

### Population control

The frequency of the detected mutations in the population was retrieved from Genome Aggregation Database (gnomAD, http://gnomad-old.broadinstitute.org/) because of its wide large-scale sequencing data. We chose the frequencies of mutation sites in all populations and in the East Asian population as controls.

### Functional prediction analysis

Candidate pathogenic mutation sites were searched in public databases, including dbSNP (https://www.ncbi.nlm.nih.gov/snp/), 1000G (https://www.internationalgenome.org/) and ExAC (The Exome Aggregation Consortium, https://exac.hms.harvard.edu). Candidate sites in HGMD (The Human Gene Mutation Database at the Institute of Medical Genetics in Cardiff, http://www.hgmd.cf.ac.uk/ac/index.php), professional version, were also searched in to determine whether pathogenicity has been reported in the literature. *PhyloP* and *PhastCons* software were used to analyse the conservation of corresponding amino acid sequence of missense mutations [11]. Pathogenic analysis was conducted by *SIFT* (http://sift-dna.org), *PolyPhen_2* (http://genetics.bwh.harvard.edu/pph2/) and *Mutation t@sting* online tools (http://mutationtaster.org/) [11–13]. We also analysed the secondary structure, disordered region and mutation effect of missense mutations by the *PredictProtein* online tool (https://predictprotein.org/). Three-dimensional structure construction of the target protein sequence was performed using *Swiss-Model* (https://swissmodel.expasy.org/) protein model structure simulation software [14]. The pathogenicity of candidate mutations was graded and judged according to the 2015 edition of the ACMG standard and guidelines [15].

## Results

The genome variation results of different patients and their families are classified and summarized based on pathogenic genes.

### Mutation distribution in patients with suspected LHON

**Fig. 1:**
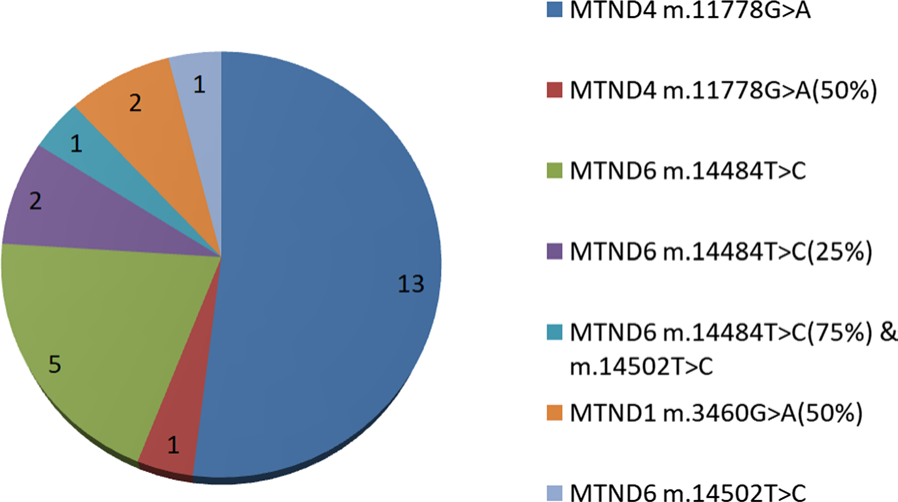
Variation distribution of 25 patients with Leber hereditary optic neuropathy (21.6%)

In total, 116 patients with suspected optic neuropathy were examined, and 25 cases of LHON were diagnosed (Fig. 1).The diagnostic rate for LHON of the Part A was 21.6% in our optic atrophy group using the mtDNA Sanger sequencing panel (*OPA1*, *WFS1*, etc., not included). The ratio of males to females among patients with LHON was 4:1 in our investigation. The average age of the patients diagnosed with LHON was 19 years old, and their age ranged from 6 to 36 years. The three common mutant sitesmt.3460, mt.11778 and mt.14484were found to be the main (96%) causes of LHON, with *MTND4* m.11778G > A being the most common pathogenic mutation, followed by *MTND6* m.14484 T > C and *MTND1* m.3460G > A. Only one rare mutant, *MTND6* m.14502 T > C, was found in these Chinese patients from central China. Several LHON patients harboured incomplete mitochondrial mutations or two mutations.

### Pathogenic mutations in hereditary retinopathy

Ninety-five families were examined by using targeted sequencing technology and were suspected to have retinitis pigmentosa or congenital retinopathy. Partial genealogical trees are depicted in Additional file 1: Figure S25. We identified 68 distinct mutations in 31 known disease genes in the patients of these families; 37 mutations are novel. The results are grouped by related genes found in retinopathy patients. In this investigation, significant mutants were detected in 57.9% of the families tested (Tables 1 and 2). The mutations listed in Table 1 are predicted to be damaging or disease causing by function prediction software, and some of the mutations have been studied and reported. The phenotypes and mutations of these families co-segregate. Targeted sequencing of retinopathy-related genes for Part B provided a 45.3% diagnostic rate, and another 12.6% of the families in this study carried candidate gene mutations with undefined pathogenicity. The diagnostic rate of RP and MD was 45.5% (30/66), and the significant detection yield was 57.6% (38/66). The diagnostic rate of multiple fundus lesions or retinal dysplasia was 44.8% (13/29), and the significant detection yield was 58.6% (17/29).

**Fig. 2:**
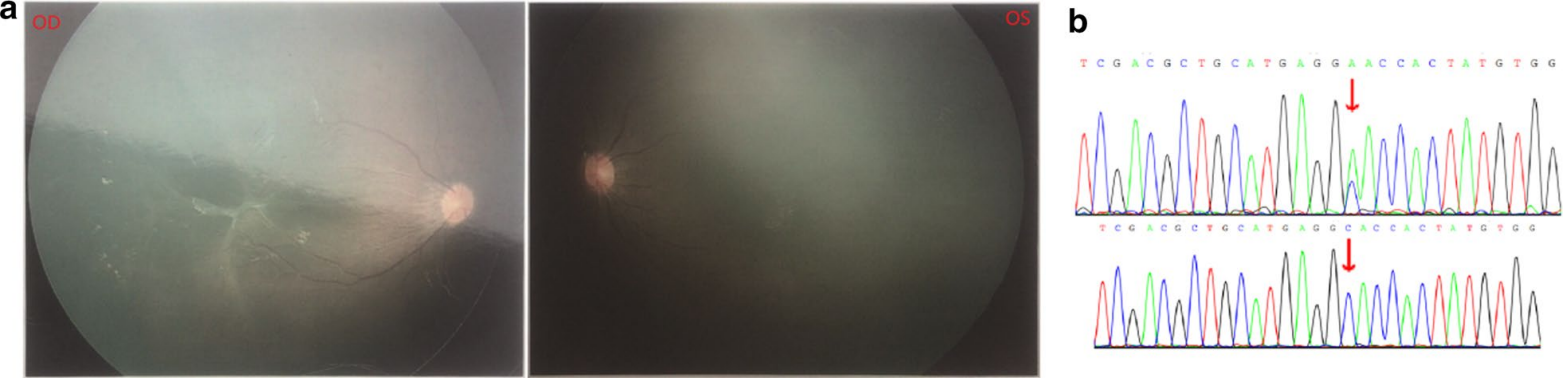
*NDP* c.124C > A hemizygous mutation and the fundus avascular area of the FEVR2 patient in Family 18. In part **a**, fundus examination of the one-year-old patient showed an avascular area in both eyes. The temporal side of the blood vessel arch in the right eye fundus showed the epiretinal membrane and macular traction. Part **b**, *NDP* c.124C > A mutation of the mother and the child, respectively

Four families (families 14, 15, 48 and 54) developed retinitis pigmentosa caused by *RHO* mutations, and the patients in these families manifested night blindness in childhood, visual field defects or tubular visual fields and retinitis pigmentosa. The Sanger sequencing results for mutations in Family 14 and Family 15 are presented in Additional file 1: Figures S13 and S14, respectively. *NDP* mutations can lead to familial exudative vitreoretinopathy (FEVR) or Norrie disease. Two families (18 and 46) with FEVR2 carried two novel *NDP* mutations, c.124C > A (p.H42N) (Fig. 2) and c.401_402delGA (p.*134Wfs*13). The eyes of those with FEVR2 do not follow movement when they are a few months old, and no blood vessel area of the binocular fundus is detected by ophthalmoscopic examination. Male patients of the two families had no other serious visual problems. Two families (32 and 55) diagnosed with Norrie disease carried two known *NDP* mutations, c.343C > T (p.R115X) and c.268C > T (p.R90C). The two-month-old male patient in Family 32 had vitreous hyperplasia, right microphthalmos and microcorneas; the male patient in Family 55 had legal blindness and atrophy of the eyeballs. Mutation of *USH2A* can cause retinitis pigmentosa with or without sensorineural hearing loss. The patients of Family 7 and Family 47 with Usher syndrome type 2A presented with retinitis pigmentosa and hearing impairment, harbouring different mutations in the *USH2A* gene (Additional file 1: Figures S11, S17 and S18). The patient of Family 9 with *USH2A* mutation had nonsyndromic retinitis pigmentosa. Three families (families 27, 38 and 51) carried different *RS1* hemizygous mutations in the retinoschisis patients. The results of fundus examination and optical coherence tomography of the patient with congenital retinoschisis in Family 38 are shown in Additional file 1: Figures S5 and S6. Patients from Family 1 and Family 2 maybe diagnosed with RP 38 caused by *MERTK* gene mutation. These cases are characterized by retinitis pigmentosa, night blindness and visual field loss.

A small deletion and nonsense mutation in *CYP4V2* was found to be the cause of the Bietti crystalline corneoretinal dystrophy of the patients from Families 3 and 4. The visual electrophysiology results for Family 3 are shown in Additional file 1: Figure S1. The *CYP4V2* c.(802–8)_810delTCATACAGGTCATCGCTinsGC and c.958C > T mutations in Family 4 are shown in FAdditional file 1: Figures S8 and S9. *FSCN2* c.72delG was the cause of RP 30 in two unrelated families (families 5 and 6). The Sanger sequencing result for *FSCN2* c.72delG is shown in Additional file 1: Figure S10. A small deletion and frameshift mutation in *PRPF31* led to RP 11 in Family 12 (*PRPF31* c.1074-8_1079delGTACCGGTCCCCAG novel mutation in Additional file 1: Figure S12) and Family 50. There were four RP patients from three generations in Family 12. In addition to the symptoms of retinitis pigmentosa, night blindness and tubular visual field, the proband and his father (Additional file 1: Figure S25) also underwent postoperative cataract extraction with intraocular lens implantation. There were two families (families 33 and 52) with a family history of RP and night blindness caused by the same mutation: *RPGR* c.2236_2237delGA. Two families (families 43 and 44) had a family history of RP and night blindness caused by different mutations of *RP2*, which included the reported splicing mutation c.769-2A > G and the novel frameshift mutation c.572_582dup11.

Seventeen families affected by different retinal diseases carried pathogenic or likely pathogenic mutations in17 different related genes. The patient of Family 36 with macular degeneration had poor eyesight. The patient of Family 16 had retinochoroidal coloboma, and his visual field examination and mutation sequencing results are provided in Additional file 1: Figure S4. Sanger sequencing results of the mutant site in Family 17 are shown in Additional file 1: Figure S15, and the RP proband also had cataracts when he was twenty-six years old. The patient of Family 20 was two years old (Sanger results in Additional file 1: Figure S20). Her full-field ERG (electroretinogram) showed that the rod cells had no waves, while scotopic ERG showed decreased amplitudes of α and β waves. The ophthalmoscopic image and sequencing results of RCS patients from Family 22 are presented in Additional file 1: Figure S19. The *CNGA1* mutations in Family 45 were validated by Sanger sequencing (Additional file 1: Figure S16). The thirty-four-year-old mother and her daughter in Family 34 had macular degeneration. The forty-one-year-old patient of Family 35 experienced retinal detachment, primary vitreous hyperplasia and FEVR, and his mother with the same *FZD4* c.612 T > A heterozygous mutation had the same manifestations. Both a thirty-three-year-old man and his mother with neurodystrophy and FEVR in Family 37 harboured the *LRP5* c.485_488delACGG heterozygous mutation. A three-year-old girl in Family 39 with congenital horizontal nystagmus had compound heterozygous variation of *SLC38A8*, and her parents were heterozygous carriers of the variant. A two-year-old boy in Family 40 had Leber congenital amaurosis (LCA), and his parents were heterozygous carriers of an *AIPL1* variant. The hemizygous *FRMD7* c.910C > T (p.R304X) mutation led to Nystagmus of the boy in Family 41, and his mother was a heterozygous carrier of the mutation. A five-year-old boy was diagnosed with LCA caused by *GUCY2D* c.3177_3178delAC homozygosity inherited from his parents.

### Variants of undetermined significance in retinopathy families

The mutations listed in Table 2 are predicted to be damaging or associated with the clinical phenotypes of the families and can be considered candidate mutations. The families included in Table 2 generally had no family history of hereditary diseases. Four families (8, 10, 23 and 53) showed different compound heterozygous mutations of *USH2A*, and the mutations were associated with the nonsyndromic retinitis pigmentosa of these patients but without obvious hearing impairment. The mutations found in the four families are likely pathogenic. A four-year-old boy in Family 19 carried a compound heterozygous mutation of *USH1C*; mutation of this gene can cause Usher syndrome-type 1C characterized as severe hearing impairment and retinitis pigmentosa. The boy with RP and night blindness had bilateral secretory otitis media, but his bilateral hearing was basically normal. He passed an TEOAE (transient evoked otoacoustic emissions) examination and DPOAE (distortion product otoacoustic emissions) test at acoustic frequencies of 1 k, 2 k, 4 k and 8 k Hz, but his left ear did not pass DPOAE at 0.5 k Hz. In addition, I-wave latency was slightly longer after 80 dBnHL short-tone stimulation in the ABR (auditory brainstem response) test, though other waves were normal. Therefore, the *USH1C* mutation is associated with these phenotypes but has undetermined significance. The other patients from different families (13, 25, 56, 24, 29 and 31) (Table 2) carried candidate gene mutations and corresponding phenotypes. The Sanger sequencing results for Family 56 are shown in Additional file 1: Figure S21 and S22. It should be noted that the *RS1* c.240G > C (p.Q80H) mutation did not co-segregate with the phenotype and genotype in Family 26.

### Specific cases

#### Family 2

**Fig. 3:**
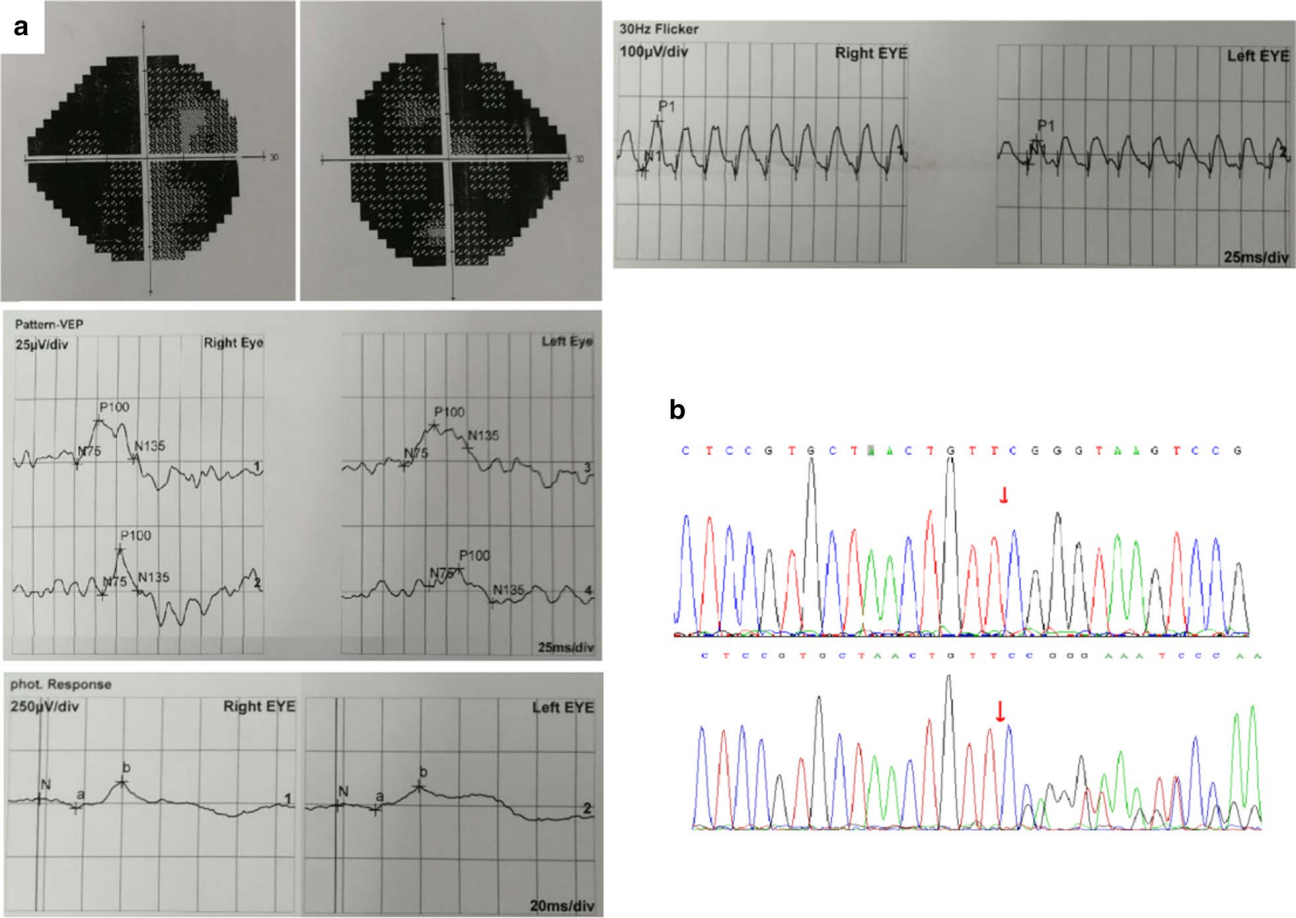
In Part **a**, P-VEP examination of the older sister with a binocular patchy visual field in Family 2 showed bilateral P100 wave latency delay with normal amplitude. F-ERG examination showed binocular light adaptation, moderately or severely decreased 30 Hz response amplitude, and moderately decreased other response amplitudes; a binocular dark response could not be induced, OPS wavelets could not be separated, other waves could be induced, and the amplitude decreased moderately. Part **b** shows the Sanger sequencing results of the mutated site

There were two RP patients in Family 2. The thirteen-year-old sister had patchy defects of the visual field and abnormal ERG, and she harboured a homozygous mutation of *MERTK* c.754delC (Fig. 3). Herten-year-old brother’s symptoms were milder, but he also had defects in the visual field (Additional file 1: Figure S7) and carried the same homozygous mutation. They all had night blindness and visual impairment. Their parents were heterozygous carriers of *MERTK* c.754delC. According to ACMG guidelines, the novel frameshift mutation of *MERTK* c.754delC should be considered pathogenic, and its grade (PVS1) is high. A healthy boy was born into this family through three generations of IVF technology (pre-implantation genetic diagnosis).

#### Family 3

The patient with homozygosity of *CYP4V2* c.(802-8)_810delTCATACAGGTCATCGCTinsGC developed retinitis pigmentosa and visual impairment. This mutation is known to be pathogenic for Bietti crystalline corneoretinal dystrophy (Bietti CCD), and it involves small deletions and insertions in splicing regions. The patient had typical fundus and visual electrophysiological symptoms (Additional file 1: Figure S24 and Figure S1). Therefore, she can be diagnosed with Bietti CCD according to ocular manifestations and gene mutations.

#### Family 5

The RP patients in Family 5 all carried the known pathogenic mutation *FSCN2* c.72delG. The proband had typical fundus and visual electrophysiological symptoms (Additional file 1: Figure S23 and Figure S2). This mutation was the same genetic cause as found for Family 6, and it is a common pathogenic mutation for RP 30.

#### Family 11

**Fig. 4:**
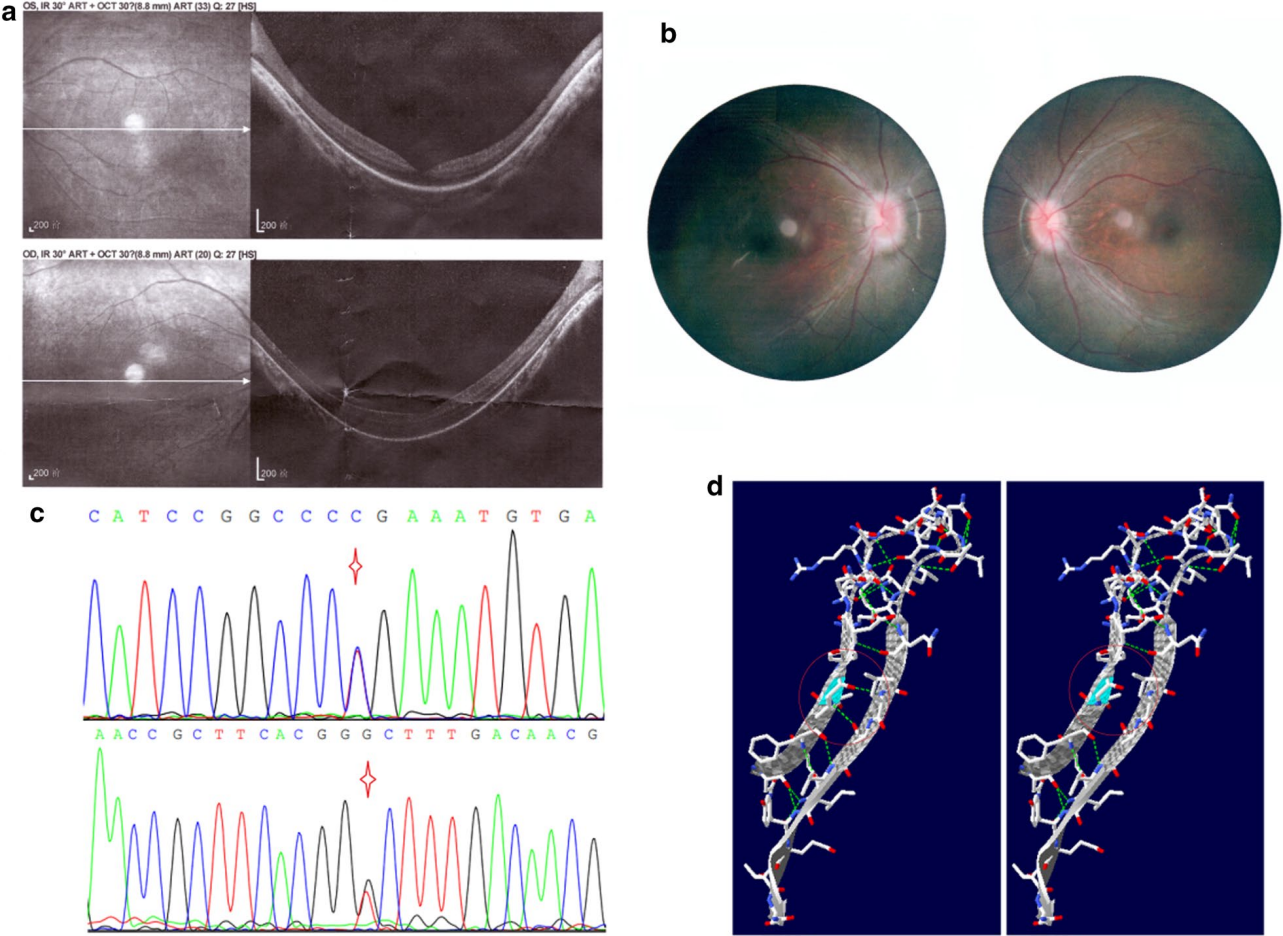
*TULP1* mutations and clinical manifestation of the LCA 15 patient in Family 11. Part **a**, optical coherence tomography (OCT) shows that the temporal retinal neuroepithelium of macula of both eyes were thinning, with the central fovea of macula forming a backward concave. Part **b**, ophthalmoscopic examination shows that the boundary of optic disc is blurred and the retina dark. Part **c**, the compound heterozygous mutation *TULP1* c.1318C > T p.R440X (above) and c.1142 T > G p.V381G (below). Part **d**, Secondary structure change of the novel mutation *TULP1* c.1142 T > G

A three-year-old boy, one of fraternal twins, was given medical advice for night blindness. The boy’s clinical manifestations also included retinal abnormalities, lateral nystagmus and finger-stimulation eyeball phenomena. He carried the *TULP1* compound heterozygous mutation c.1318C > T (p.R440X) and c.1142 T > G (p.V381G); his parents are heterozygous carriers of each of the mutations. The nonsense mutation c.1318C > T (p.R440X) is known to be pathogenic for LCA, type 15, and the missense mutation c.1142 T > G (p.V381G) is novel. c.1142 T > G can lead to amino acid substitution and affects the protein’s function. The OCT (optical coherence tomography) image, fundus photography and mutations are presented in Fig. 4. The boy was diagnosed with LCA 15 according to his clinical manifestations and gene mutations.

#### Family 18

The one-year-old boy’s fundus photographs and mutation sequencing results are shown in Fig. 2. The cornea of both eyes was clear, the anterior chamber was preserved, and the lens was transparent. Fundus photography showed no blood vessel area in either eye. The temporal epiretinal membrane of the right fundus vascular arch pulled the macula. *NDP* mutation can lead to FEVR2, and c.124C > A (p.H42N) is a novel mutation causing FEVR2. There is one known pathogenic mutation of c.125A > G (p.H42R) at the same location of the polypeptide chain of this novel variant. According to ACMG guidelines and related prediction software, c.124C > A (p.H42N) should be pathogenic. FEVR2 is characterized by no blood vessel area of the fundus, but the severity of the disease varies. There three persons with c.124C > A (p.H42N) mutation in this family showed no blood vessel area in either fundus.

#### Family 21

**Fig. 5:**
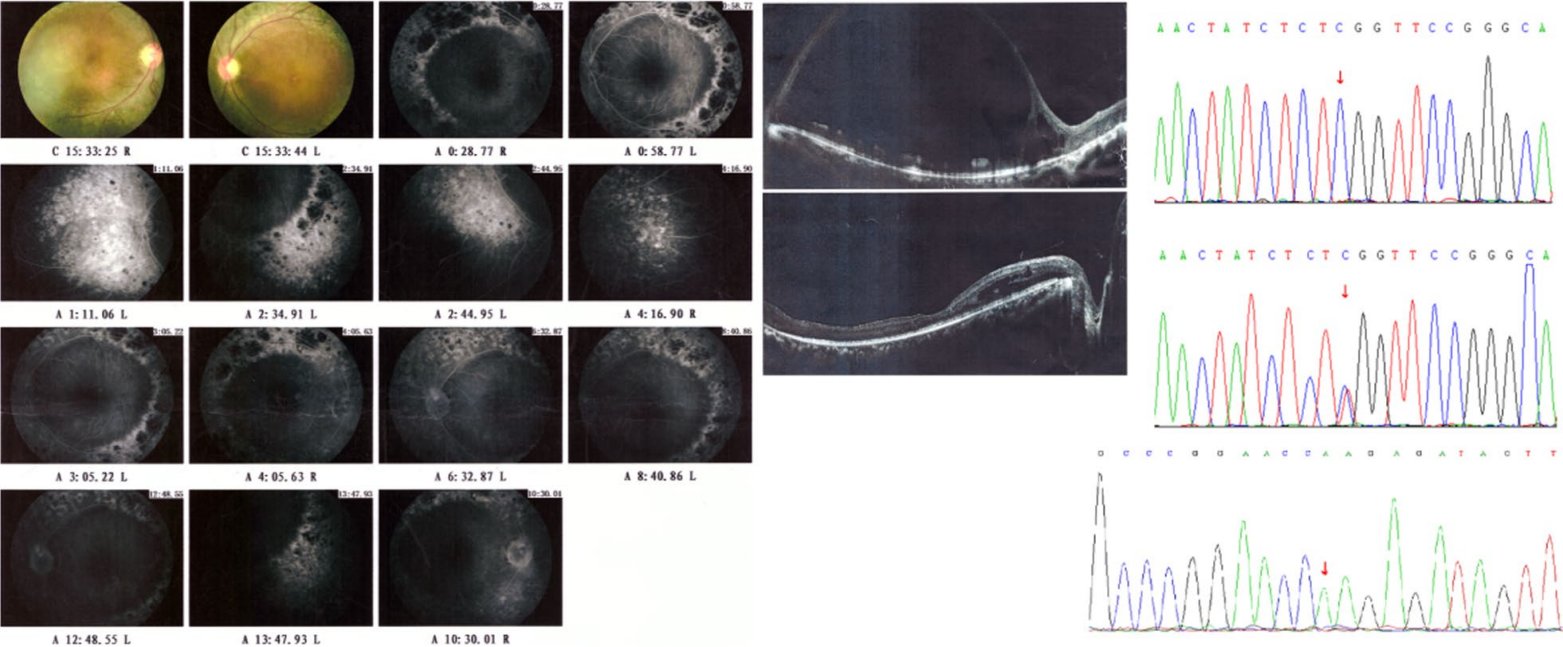
Fluorescein fundus angiography examination of the patient in family 21 showed prolonged filling time of bilateral arteriovenous fluorescence. In the early stage, inhomogeneous strong fluorescence and occluded fluorescence was seen in the posterior pole of both eyes. Strong fluorescence and inhomogeneous fluorescence was also seen in the periphery. In the late stage, inhomogeneous strong fluorescence was seen in the periphery of both eyes, and no obvious fluorescence leakage was observed.OCT examination showed a split nerve cortex in the macular area of the left eye, the fovea in the macular area were not seen, and the pigmented epithelium was rough; the fovea in the macular area of the right eye were not obvious, the pigmented epithelium in the macular area was rough, and the nasal retinal layer was split

This family of Chinese Hui nationality (a Chinese minority) involved a consanguineous marriage. The patient presented with retinoschisis, macular oedema and night blindness, and was a homozygous carrier of *NR2E3* c.925C > T (p.R309W). The ophthalmological examination and mutation sequencing results of the patient are shown in Fig. 5 and Additional file 1: Figure S3. The missense mutation c.925C > T of *NR2E3* is a novel mutation for Goldmann-Favre syndrome, but the c.925C > G (p.R309G) at the same location of mRNA and polypeptide chain is known to be pathogenic for Goldmann-Favre syndrome and enhanced S-cone syndrome [16]. Some scholars believe that Goldmann-Favre syndrome is the severe type of enhanced S-cone syndrome [17]. The patient's condition worsened over the past 10 years, and he was diagnosed with Goldmann-Favre syndrome according to his phenotype and genotype.

## Discussion

Using targeted NGS technology and Sanger sequencing, we investigated the mutation profile and clinical features of 211 Chinese families with hereditary retinopathy over three years. Ninety-five families were evaluated by targeted next-generation sequencing, and fifty-five had meaningful positive findings. One hundred and sixteen patients from different families were tested by Sanger sequencing, and twenty-five members carried related mitochondrial mutations. Hereditary retinopathy covers a group of genetically and clinically highly heterogeneous disorders [1]. Targeted NGS analysis is a valuable method for molecular genetics diagnostics of these diseases, as supported by previous studies [18–20]. These studies show that the potential molecular genetics diagnostic rate of targeted sequencing is between 38% [19] and 76% [20]. Jespersgaard's report indicated a detection rate of related genotypes of 72%, whereas the detection rate of causative variants was 48% [18]. Our study attained a 45.3% potential diagnostic rate of hereditary retinopathy families and a 58% meaningful detection rate of families. The diagnostic rate of the genetic tests in this study is in the middle of the range in Europe [19, 20]. Although our detection rate was lower than that is a Japanese study [18], we implemented strict standards to achieve diagnosis. Our research results also support that DNA sequencing is a powerful diagnostic tool for hereditary retinal disease.

Twenty-five patients with positive mitochondrial gene test results in our study were 19 years old on average, with a male-to-female ratio of 4:1. This indicates that retinopathy patients in the Chinese population have a younger age and higher sex ratio than those in Europe and America [21, 22]. However, our study did not find new mitochondrial variants and showed that mt.11778 and mt.14484 are the most common pathogenic mutations for LHON. The possibility of LHON diagnosis over representation may be due to the small scale and single-centre collection.

Due to clinical heterogeneity, many subjects did not have a definite ophthalmological diagnosis before NGS examination; thus, we subdivided them into two larger subtypes: multiple fundus lesions or retinal dysplasia. Overall, our genetic test results may help ophthalmologists make diagnoses or even indicate unobserved lesions confirmed by further clinical examination. Our impression is a high positive rate of genetic testing for rare and severe ocular lesions in this study. One recent study of visual impairment gene detection in a large Dutch cohort provided meaningful information, including various types of inherited eye disorders [23]. Four main types, RP, cataract, developmental eye defects and optic atrophy, were investigated in this previous research, and the detection rates were 63%, 50%, 33% and 17%, respectively. Due to imbalance in the number of subjects with different types, the detection rate of several types with few subjects may need more independent analysis. In contrast, we focused more on the genetic variation of fundus lesions such as RP, MD and specialoptic atrophy.

Retinitis pigmentosa is a hereditary progressive retinopathy. It is the most common blinding disease and is characterized by nocturnal blindness and progressive visual field defects caused by degeneration of retinal photoreceptor cells and pigment epithelial cells [24]. Its inheritance modes include autosomal dominant, autosomal recessive and X-linked recessive inheritance. Thirty families (families 14, 15, 48, 54, 7, 9, 47, 1, 2, 3, 4, 5, 6, 12, 50, 33, 52, 43, 44, 36, 17, 45, 8, 10, 23, 53, 19, 13, 24 and 31) with RP (or MD) had positive meaningful findings of gene mutations. With the help of targeted NGS, these patients were diagnosed with various types of retinitis pigmentosa. In some of these families, healthy offspring were born through genetic prenatal diagnosis or third-generation test-tube infant technology (pre-implantation genetic diagnosis). RP accounted for a large proportion of hereditary retinopathy in our study. Overall, it is difficult to distinguish cone-rod dystrophy from retinitis pigmentosa only through ophthalmic examination because of the similarity in clinical manifestations [25]. Molecular genetic tests help in making accurate diagnoses for patients with cone-rod dystrophy (Family 34). Moreover, in some patients, it is difficult to differentiate choroideremia from RP, and the detection of *CHM* gene mutations has noteworthy diagnostic value. Choroideremia has a worse prognosis than RP [26], and the patient of Family 16 with choroideremia was diagnosed through targeted sequencing. Congenital stationary night blindness (CSNB) is similar to RP in clinical presentation, but its prognosis is better. We identified a case (Family 20) of CSNB, type 1C, through genetic targeting sequencing in this study.

Vitreoretinopathy is another major type of hereditary retinopathy. In nine families (families 18, 32, 46, 55, 35, 37, 49, 25, 56), gene mutations related to such diseases were detected. FEVR is a retinal vascular structural abnormality with different inheritance patterns. The clinical symptoms of the disease vary greatly, even in the same family [27]. For example, mild cases have no symptoms; the only disease-related abnormality is a circular arc without vascular retina at the periphery of the terminal temporal area. These characteristics were observed in this study. Non-syndromic retinoschisis is an X-linked hereditary retinopathy, and its known pathogenic gene is *RS1* [28]. We observed three different known pathogenic mutations of *RS1* in three different families (Families 27, 38 and 51). The new mutation *RS1* c.240G > C found in Family 26 may be benign because both the patient and his normal maternal grandfather carried it. Goldmann-Favre syndrome is an ocular syndrome with clinical symptoms, including retinoschisis (Family 21).

LCA involves early onset and serious impairment of visual function [29]. Most children become blindness. Parents can usually observe visual abnormalities within one year of the child's birth. Children from four different families (families 11, 28, 40 and 42) were diagnosed with LCA in our investigation, showing that targeted sequencing is of great significance for the diagnosis of hereditary ophthalmopathy and that it will become part of our eye health management. We also detected two cases of fundus developmental disease: renal coloboma syndrome (Family 22) and foveal hypoplasia (Family 39). These two diseases are general lyuntreatable and have a general prognosis, but families with the disease might avoid high-risk offspring according to genetic rules. Two families (families 41 and 29) with nystagmus carried two different mutations, *GPR143* and *FRMD7*. *GPR143-*or*FRMD7*-related nystagmus shows X-linked inheritance, with or without obvious retinal abnormalities [30]. The genetic causes can guide these two families in having healthy offspring.

Previous studies have used the Sanger method to sequence only one or several genes for the molecular diagnosis of patients with different retinal diseases [31–34]. One Bietti crystalline corneoretinal dystrophystudy showed an 84% detection rate by *CYP4V2* sequencing alone [31], but it is likely to be a single example of over representation due to the only known pathogenic gene being *CYP4V2*. The related detection rates of two FEVR studies in which three and six genes were sequenced were just 23% [32] and 38.7% [33] respectively. Meaningful results of genetic testing usually require a high degree of accurate clinical diagnosis. Nevertheless, retinal diseases have complex clinical manifestations and genetic heterogeneity. The clinical symptoms of some diseases are difficult to distinguish, and some diseases are related to multiple genes. As the number of genes needed to be detected increases, the efficiency of the Sanger sequencing method decreases, and targeted sequencing becomes a better choice. Analysis of exon copy number variants in targeted gene was also executed in this study using panel sequencing data, though there were no positive findings. Exon duplication of *OCRL* was found in Lowe syndrome in our previous work [35].

In summary, we report 37 novel related meaningful mutations and 31 known pathogenic variants for retinopathy in 31 different genes, leading to different relevant phenotypes of eye diseases. The diagnostic rate of LHON was 21.6% in our study, but no new mitochondrial pathogenic mutations were found. To our knowledge, this is a larger-scale medical genetic study of retinal diseases in the Chinese population than previously reported. The innovation of this research is that we report new variants and phenotypes of diseases as well as the important role of sequencing results in diagnosis and differential diagnosis. New research advances suggest that molecular genetic tests may be used not only to clarify diagnoses and to direct counselling but also to move the field of 'incurable' and 'blinding' inherited retinal diseases substantially forward [1]. Our study demonstrates the importance of examining a large collection of families with hereditary retinopathy because of the clinical manifestations and genetic heterogeneity of the diseases, with guiding significance for this disease diagnosis and aristogenesis.

## Conclusion

The targeted NGS of the human genome in related Chinese families in this study expands the mutational spectrum and deepens our understanding of the mechanism of disease. This investigation also increases knowledge of the heterogeneity of clinical manifestations of diseases and enriches the phenotypic spectrum of diseases. Our study contributes novel mutations and the phenotypic aspects of retinopathy and reveals the genetic and clinical heterogeneity of related conditions. Our results illustrate the significance of molecular genetic testing for patients with hereditary retinopathy.

## Supplementary Information

**Additional file 1**. Supplementary material of tables and figures.

## Data Availability

The raw datasets used and analysed during the current study are not deposited in publicly available repositories because of considerations about the security of human genetic resources. The transcript RefSeq number (Tables 1 and 2) was obtained from the Ensembl database (http://asia.ensembl.org) [10]. Any questions should be directed to the corresponding author. We provide conclusive variant information without identifying/confidential patient data in the paper or its appendix. For other details of the availability of data and material, please refer to the methods section of the article.

## Abbreviations

NGS: Next-generation sequencing
gnomAD: Genome Aggregation Database
dbSNP: The Single Nucleotide Polymorphism Database
1000G: The 1000 Genomes Project
ExAC: The Exome Aggregation Consortium
HGMD: The Human Gene Mutation Database at the Institute of Medical Genetics in Cardiff
ACMG: The American College of Medical Genetics and Genomics
LHON: Leber hereditary optic neuropathy
RP: Retinitis pigmentosa
MD: Macular degeneration
