# Supplementary material for: Genetic investigation of 211 Chinese families expands the mutational and phenotypical spectra of hereditary retinopathy genes through targeted sequencing technology
Source: BMC Med Genomics. 2021 Mar 29;14:92. doi: 10.1186/s12920-021-00935-w (PMC8008643; doi:10.1186/s12920-021-00935-w)
Supplement: Supplementary file 1 — Additional file 1. Supplementary material of tables and figures. [file 12920_2021_935_MOESM1_ESM.docx]

SupplementaryMaterial

Table S1. GenelistoftheNGSpenal

| ABCA4 | ABCB6 | ABCC6 | ABCC6P2 | ABHD12 | ACBD5 | ACE | ACO2 |
| --- | --- | --- | --- | --- | --- | --- | --- |
| ADAM9 | ADAMTS18 | ADAMTSL4 | ADGRA3 | ADGRV1 | ADIPOR1 | AGBL5 | AHI1 |
| AHR | AIPL1 | AKT3 | ALDH1A3 | ALDH3A2 | ALMS1 | ANGPT2 | ANGPT4 |
| ANO5 | AP3B1 | AP3D1 | APOE | ARHGEF18 | ARL13B | ARL2BP | ARL3 |
| ARL6 | ARMC9 | ARMS2 | ARSG | ASRGL1 | ATF6 | ATOH7 | ATXN7 |
| B9D1 | B9D2 | BAP1 | BBIP1 | BBS1 | BBS10 | BBS12 | BBS2 |
| BBS4 | BBS5 | BBS7 | BBS9 | BCL10 | BEST1 | BHLHE23 | BLOC1S3 |
| BLOC1S6 | BMP4 | C1QTNF5 | C2 | C3 | C5AR2 | C8orf37 | C9 |
| CA4 | CABP4 | CACNA1F | CACNA2D4 | CAPN5 | CASR | CC2D2A | CCDC28B |
| CCT2 | CDH23 | CDH3 | CDHR1 | CEP104 | CEP120 | CEP164 | CEP19 |
| CEP250 | CEP290 | CEP41 | CEP78 | CERKL | CFAP410 | CFB | CFH |
| CFHR1 | CFHR3 | CFHR4 | CFI | CHM | CIB2 | CLDN19 | CLN3 |
| CLN5 | CLN6 | CLN8 | CLRN1 | CLUAP1 | CNGA1 | CNGA3 | CNGB1 |
| CNGB3 | CNNM4 | CNOT9 | COL11A1 | COL11A2 | COL18A1 | COL2A1 | COL4A1 |
| COL8A1 | COL9A1 | COL9A2 | CPLANE1 | CRB1 | CRX | CSN3 | CSPP1 |
| CST3 | CTC1 | CTNNA1 | CTNNB1 | CTNS | CTSD | CTSF | CWC27 |
| CX3CR1 | CYP4V2 | DHCR7 | DHDDS | DHX38 | DMD | DNAJC5 | DPP3 |
| DR1 | DRAM2 | DTHD1 | DTNBP1 | EFEMP1 | ELOVL4 | EMC1 | ENPP1 |
| ERCC2 | ERCC3 | ERCC4 | ERCC5 | ERCC6 | ERCC8 | EXOSC2 | EYA1 |
| EYS | FAM161A | FBLN5 | FBN2 | FKRP | FKTN | FLVCR1 | FSCN2 |
| FXN | FZD4 | GDF3 | GDF6 | GGCX | GMPPB | GNAT1 | GNAT2 |
| GNB3 | GNPTG | GPR143 | GPR179 | GPR34 | GRK1 | GRM6 | GRN |
| GUCA1A | GUCA1B | GUCY2D | GUSB | HARS | HEXA | HEXB | HFE |
| HGSNAT | HK1 | HMCN1 | HMX1 | HPS1 | HPS3 | HPS4 | HPS5 |
| HPS6 | HSPA5 | HTRA1 | IDH3B | IDUA | IFT140 | IFT172 | IFT27 |
| IFT43 | IFT74 | IFT81 | IGFBP7 | IMPDH1 | IMPG1 | IMPG2 | INPP5E |
| INVS | IQCB1 | ITGA2B | ITGB3 | ITM2B | JAG1 | KCNJ13 | KCNV2 |
| KIAA0556 | KIAA0586 | KIAA1549 | KIF11 | KIF7 | KIZ | KLHL7 | LAMA1 |
| LARGE1 | LCA5 | LRAT | LRIT3 | LRMDA | LRP2 | LRP5 | LRP6 |
| LRPAP1 | LYST | LZTFL1 | MAK | MAN2B1 | MANBA | MAPKAPK3 | MC1R |
| MCOLN1 | MERTK | MFN2 | MFRP | MFSD8 | MIR204 | MITF | MKKS |
| MKS1 | MLPH | MPDZ | MTHFR | MTTP | MVK | MYO5A | MYO7A |
| NAT8 | NBAS | NDP | NEK2 | NEUROD1 | NINL | NMNAT1 | NPHP1 |
| NPHP3 | NPHP4 | NR2E1 | NR2E3 | NR4A3 | NRL | NUB1 | NXNL1 |
| NYX | OAT | OCA2 | OFD1 | OPA1 | OPA3 | OPN1LW | OPN1MW |
| OPN1MW2 | OPN1SW | OR2W3 | OTX2 | P3H2 | PANK2 | PAX2 | PAX6 |
| PCARE | PCDH15 | PCYT1A | PDCD2 | PDE6A | PDE6B | PDE6C | PDE6D |
| PDE6G | PDE6H | PDZD7 | PEX1 | PEX10 | PEX11B | PEX12 | PEX13 |
| PEX14 | PEX16 | PEX19 | PEX2 | PEX26 | PEX3 | PEX5 | PEX5L |
| PEX6 | PEX7 | PGK1 | PGR | PHYH | PIBF1 | PITPNM3 | PLA2G5 |
| PLEKHA1 | PLK4 | PMPCA | PNPLA6 | POC1B | POC5 | POMGNT1 | POMT1 |
| POMT2 | PON1 | PON3 | PPT1 | PRCD | PRDM13 | PROM1 | PRPF3 |
| PRPF31 | PRPF4 | PRPF6 | PRPF8 | PRPH2 | PRPS1 | PRSS56 | RAB27A |
| RAB28 | RABGGTA | RABGGTB | RAX | RAX2 | RB1 | RBM33 | RBP3 |
| RBP4 | RCBTB1 | RD3 | RDH11 | RDH12 | RDH5 | REEP6 | RGR |
| RGS9 | RGS9BP | RHO | RIMS1 | RLBP1 | ROM1 | RP1 | RP1L1 |
| RP2 | RP9 | RPE65 | RPGR | RPGRIP1 | RPGRIP1L | RS1 | SAG |
| SALL2 | SAMD11 | SCO2 | SDCCAG8 | SEMA4A | SGCD | SHH | SHOX |
| SIX5 | SIX6 | SLC19A2 | SLC24A1 | SLC24A5 | SLC25A15 | SLC26A4 | SLC38A1 |
| SLC38A8 | SLC39A5 | SLC45A2 | SLC7A14 | SNRNP200 | SOD1 | SOD2 | SPATA7 |
| SPP2 | STRA6 | SUFU | TBK1 | TCF7L2 | TCTN1 | TCTN2 | TCTN3 |
| TEAD1 | TENM3 | TIMP3 | TINF2 | TLR3 | TLR4 | TLR6 | TMEM107 |
| TMEM126A | TMEM138 | TMEM216 | TMEM231 | TMEM237 | TMEM67 | TMEM98 | TOPORS |
| TPP1 | TRAF3IP1 | TREX1 | TRIM32 | TRNT1 | TRPM1 | TSPAN12 | TTC21B |
| TTC8 | TTLL5 | TTPA | TTR | TUB | TUBB4B | TUBGCP4 | TUBGCP6 |
| TULP1 | TYR | TYRP1 | UNC119 | USH1C | USH1G | USH2A | VCAN |
| VEGFA | VHL | VPS13B | VSX2 | WDPCP | WDR19 | WFS1 | WHRN |
| XYLT1 | XYLT2 | YAP1 | ZNF408 | ZNF423 | ZNF513 | ZNF644 | - |

Table S2. Primer pairs of the mutation hotspot of *LHON*

| Gene | Upstream sequence (5’->3’) | Downstream sequence (5’->3’) | Mutant sites |
| --- | --- | --- | --- |
| ***MTND1*** | CGCCTTCCCCCGTAAATGATATC | GGGGGTTCGGTTGGTCTCTG | **mt.3460**, mt.3376, mt.3635, mt.3700, mt.3733 |
| ***MTND4*** | CCCCTGACAAAACACATAGCCTAC | GGGGGCATGAGTTAGCAGTTC | **mt.11778** |
| ***MTND6*** | TGGGGAGGTCGATGAATGAGT | CCCCCGCACCAATAGGATC | **mt.14484**, mt.14482, mt.14495, mt.14502, mt.14568, mt.14498, mt.14325 |


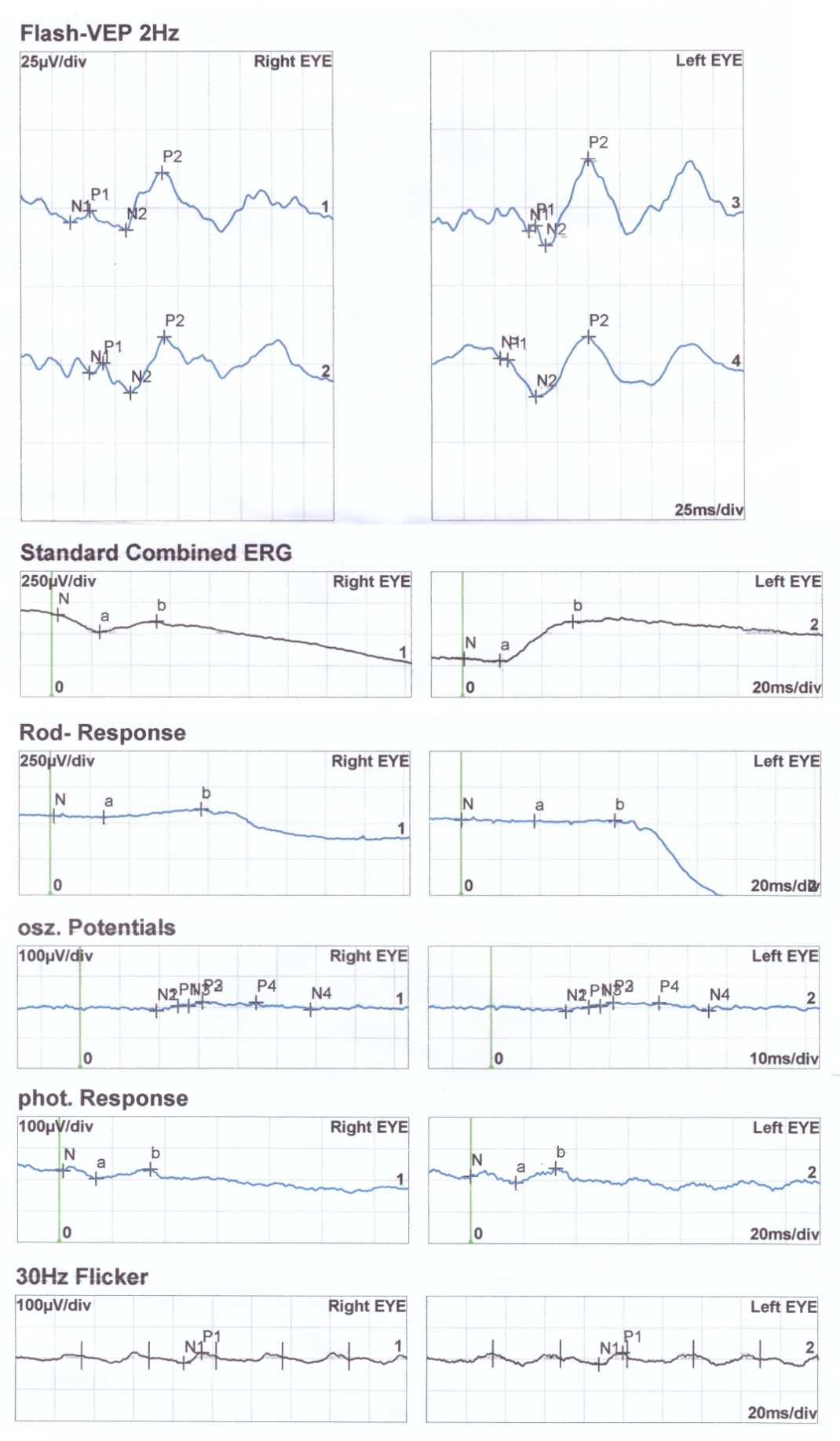


Figure S1. The visual electrophysiology examination of patient in Family 3. F-VEP shows that the latency and amplitude of P2 wave in the right eye were normal, while the latency of P2 wave in the left eye was slightly delayed and the amplitude was normal. F-ERG shows that each wavelet of the binocular OPS response was poorly differentiated, and the amplitudes of the other response were moderately and severely decreased.


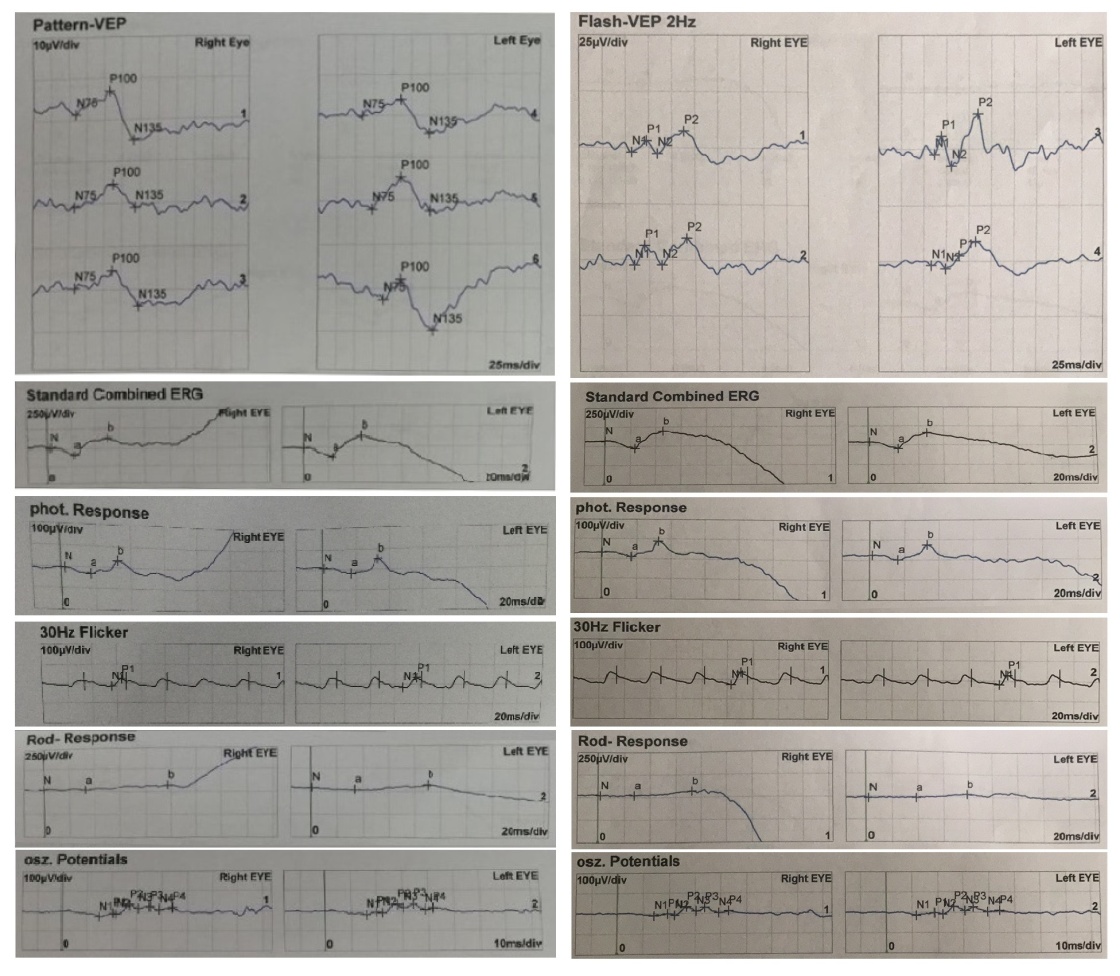


Figure S2. The examination of visual electrophysiology of patient in Family 5. P-VEP examination showed that the latency of P100 wave in both eyes was delayed and the amplitude was decreased. F-ERG examination showed that binocular rod response and 30Hz response amplitude decreased moderately or severely, other response amplitudes decreased moderately or severely.


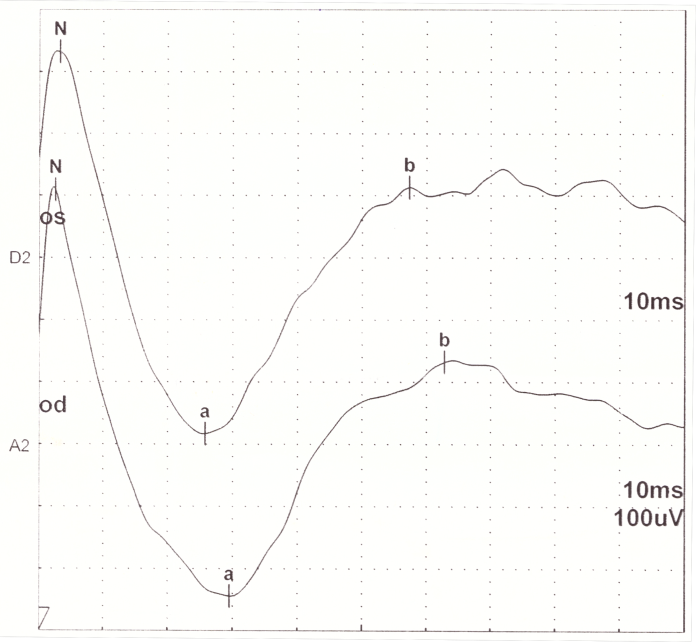


Figure S3. The result of flash electroretinogram of patient in Family 21 showed that the amplitude of a and b wave was normal, the latency of a and b wave was prolonged, and the wave oscillating potential (ops) was increased.


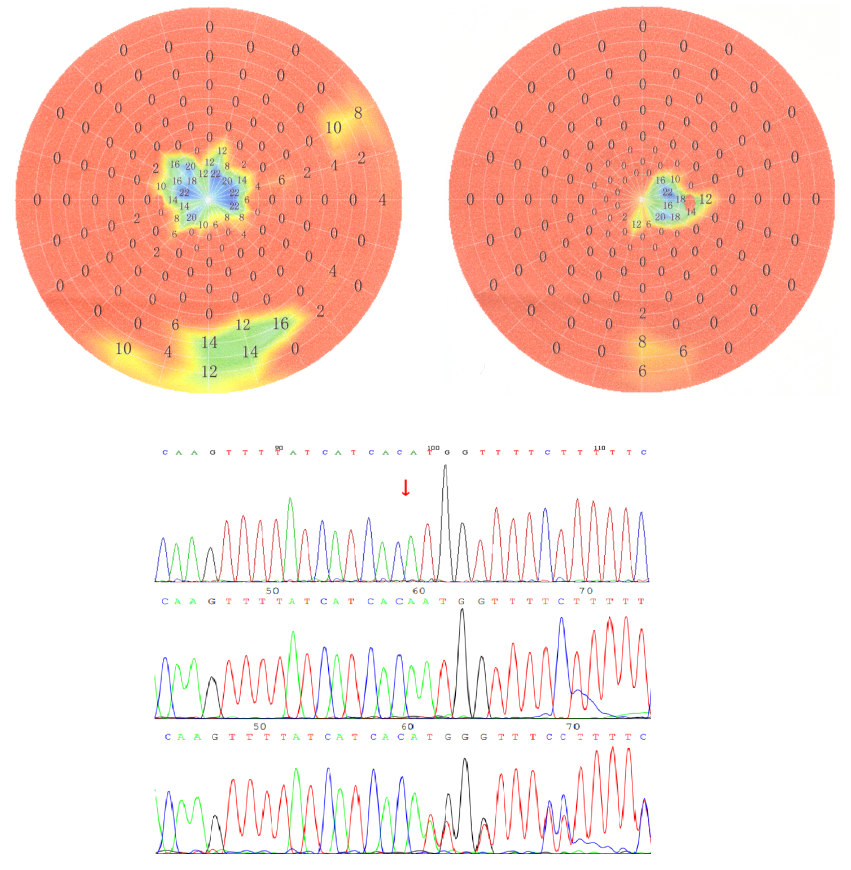


Figure S4. Visual field and Sanger sequencing of the mutation sites of the patient in Family 16

Left eye: residual visual field around the upper and lower nose, central residual visual field;

Right eye: residual visual field around the lower nose, central residual visual field


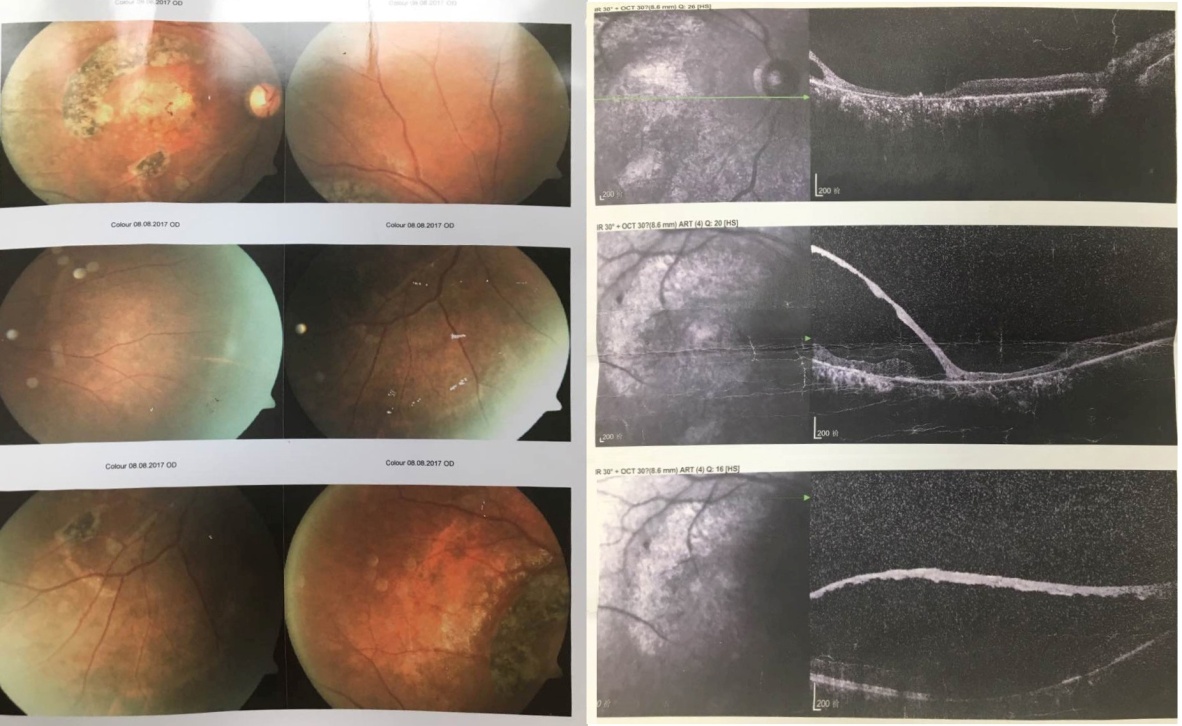


Figure S5. Fundus examination and OCT of right eye of the congenital retinoschisis patient in Family 38.


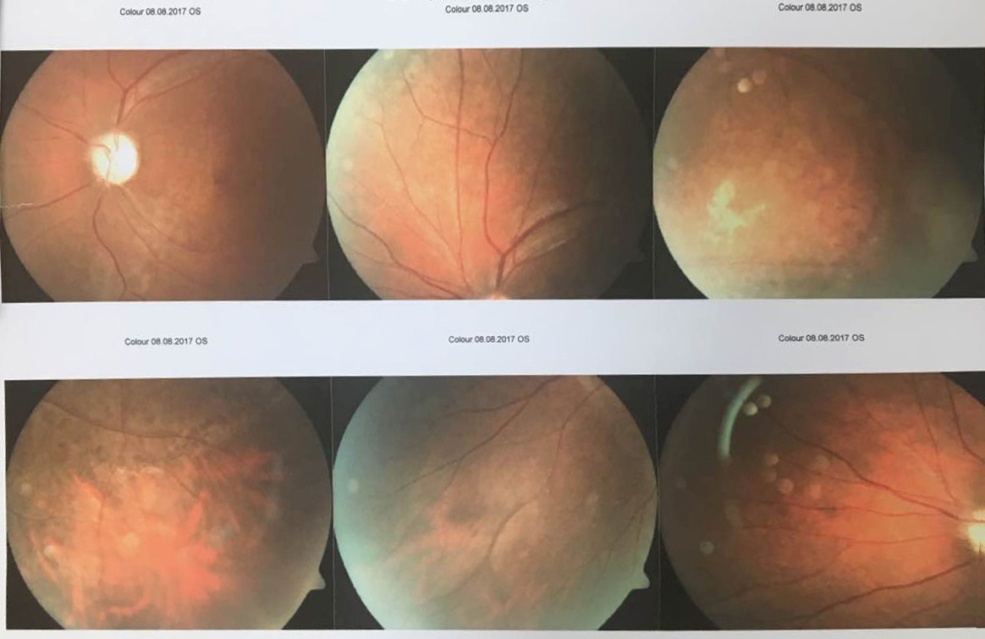


Figure S6. Fundus examination of left eye of the congenital retinoschisis patient in Family 38.


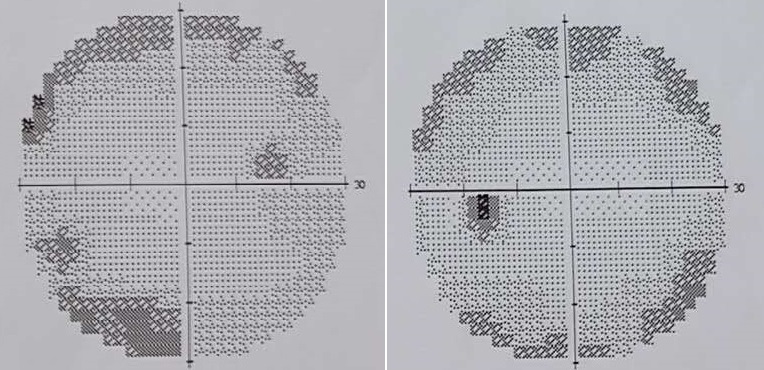


Figure S7. The younger brother with decreased binocular visual field in Family 2.


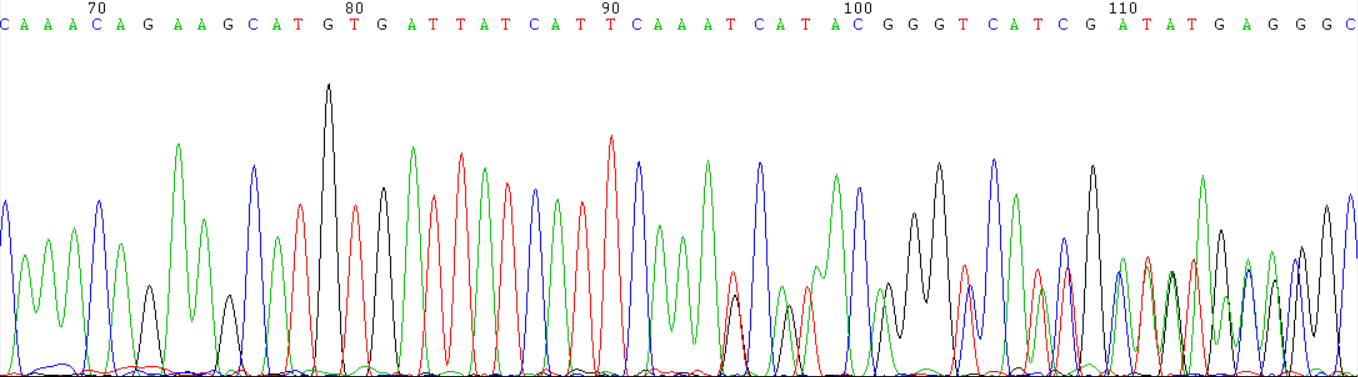


Figure S8. The *CYP4V2* c.(802-8)_810delTCATACAGGTCATCGCTinsGC mutation in Family 4


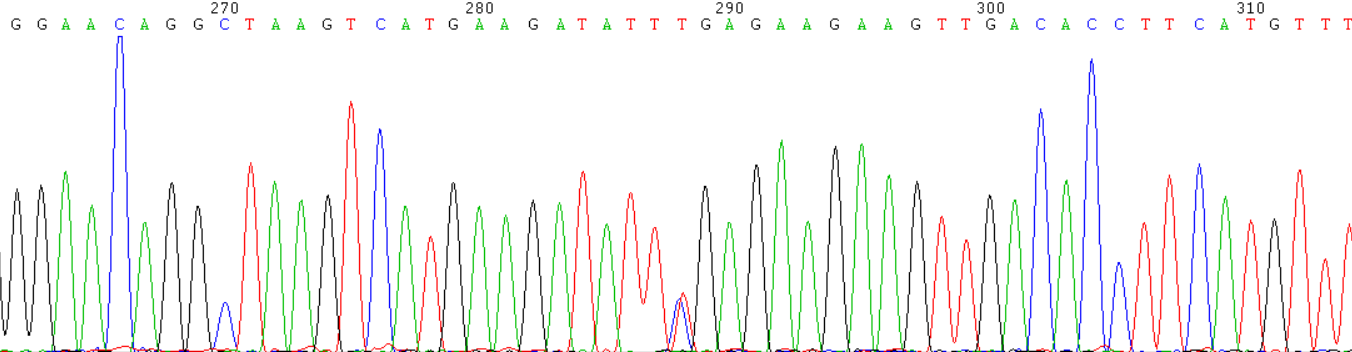


Figure S9. The *CYP4V2* c.958C>T mutation in Family 4


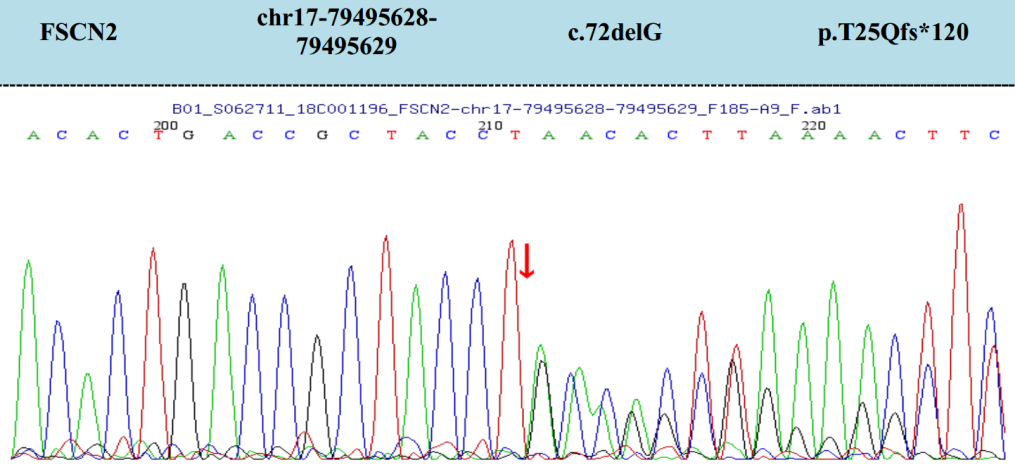


Figure S10. The *FSCN2* c.72delG mutation of patient in Family 6


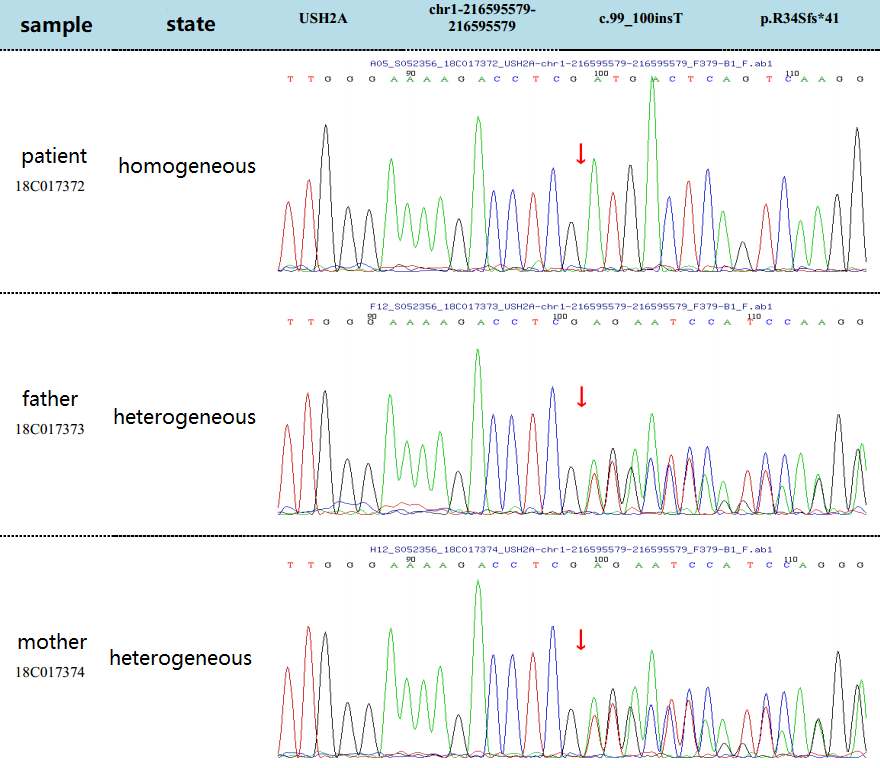


Figure S11. The *USH2A* c.99_100insT mutation in Family 7


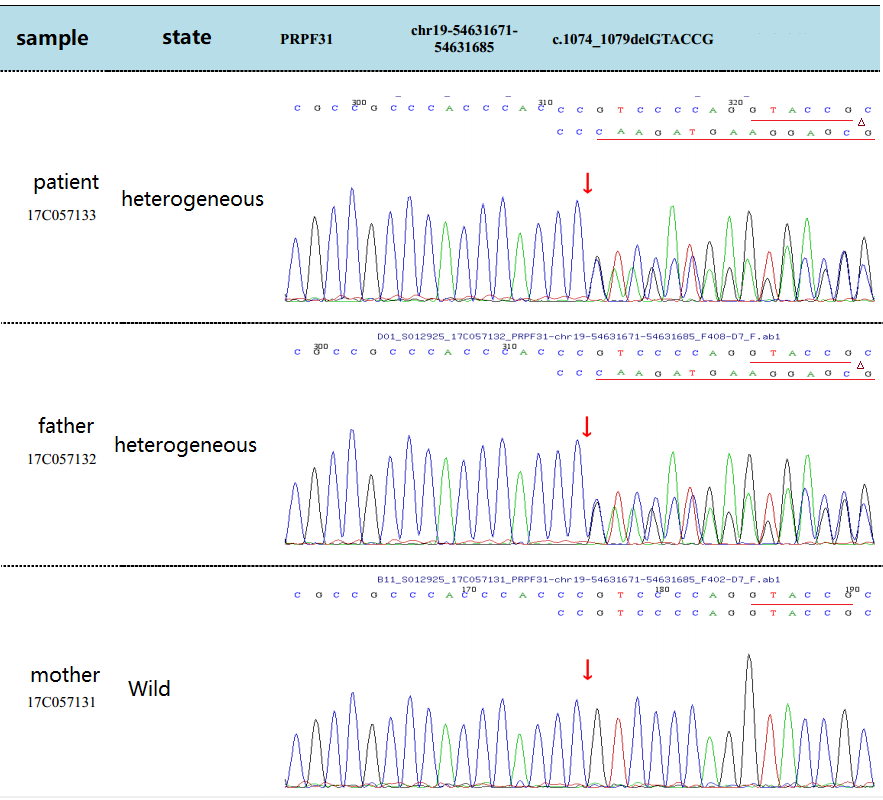


Figure S12. The *PRPF31* c.1074-8_1079delGTACCGGTCCCCAG mutation in Family 12


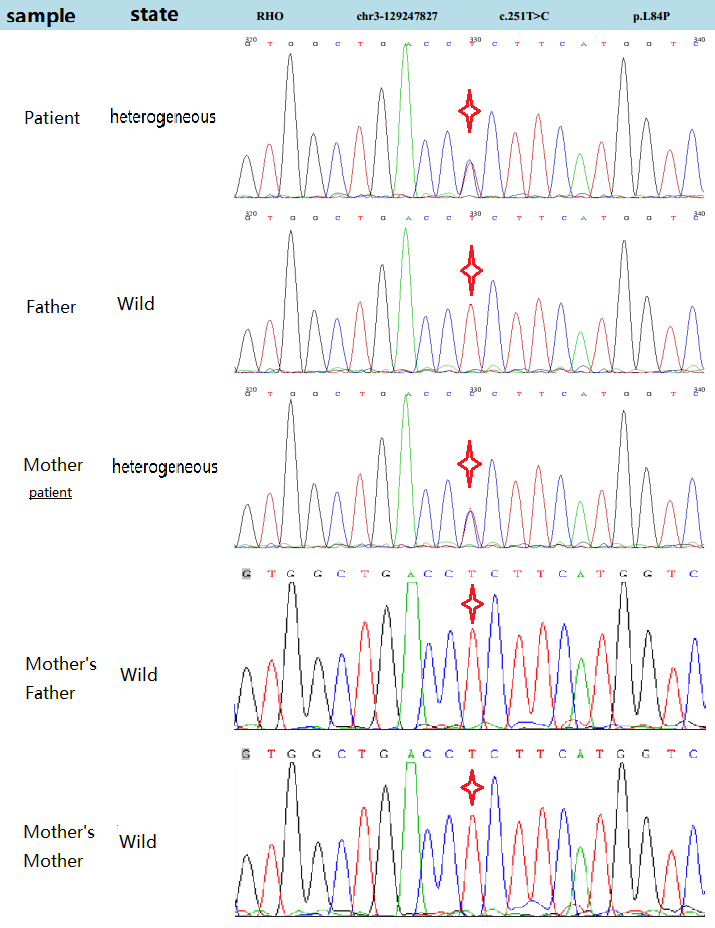


Figure S13. The *RHO* c.251T>C mutation in Family 14


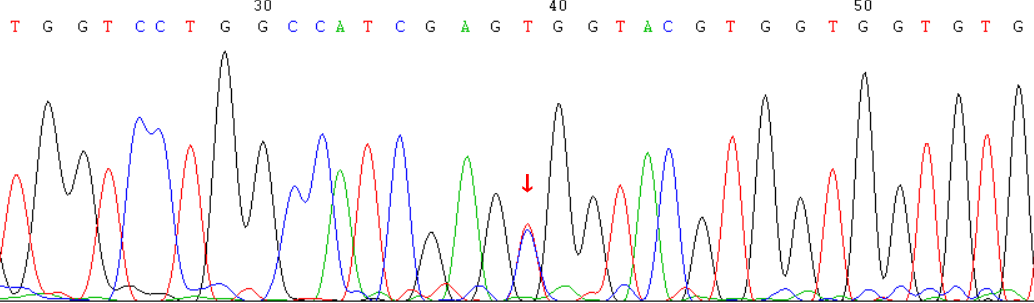


Figure S14. The *RHO* c.403C>T mutation in Family 15


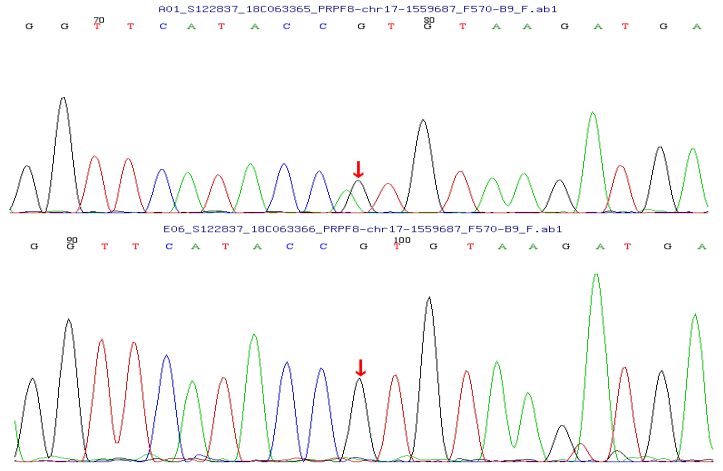


Figure S15. The *PRPF8* c.5792C>T mutation in Family 17


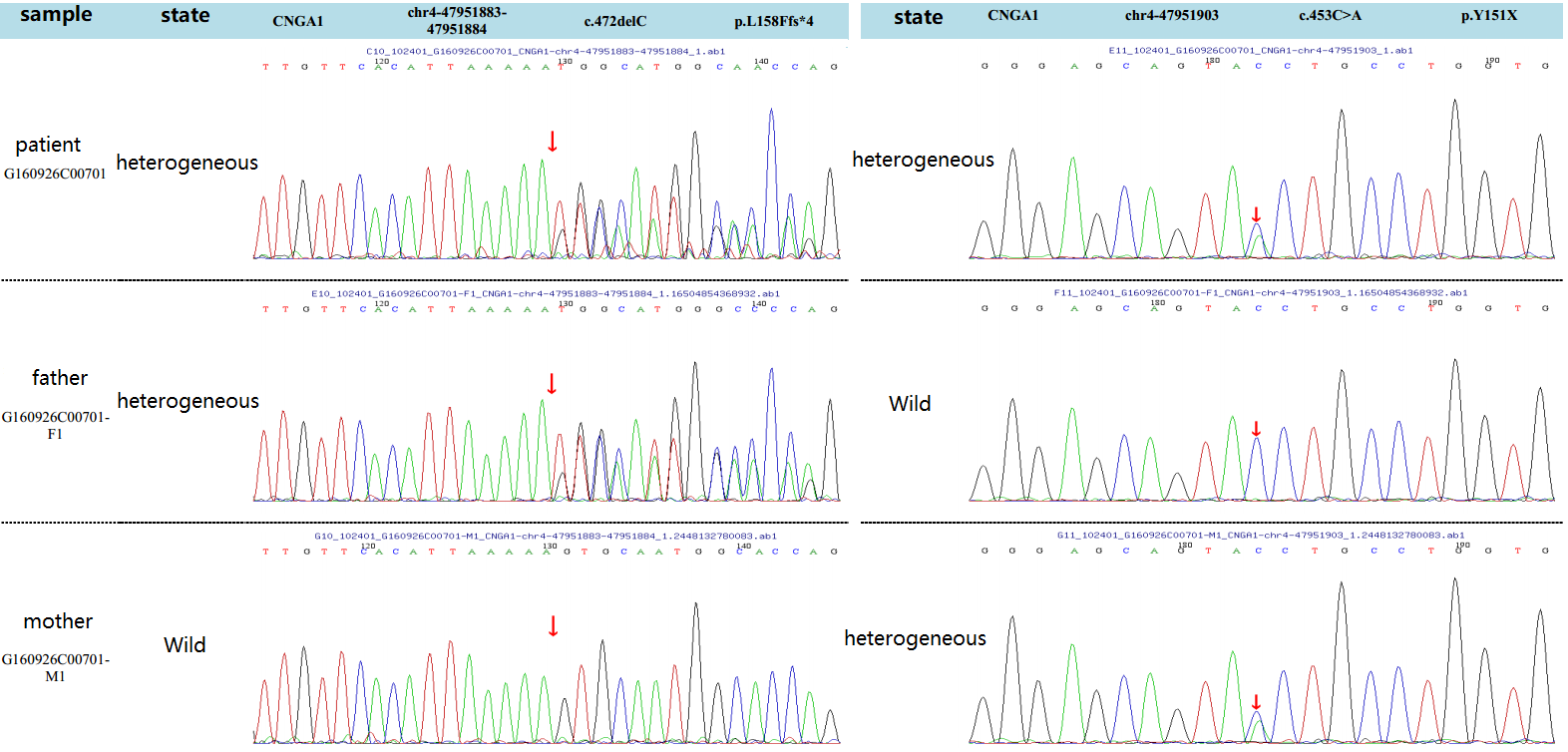


Figure S16. The *CNGA1* c.472delC and c.453C>A mutation in Family 45


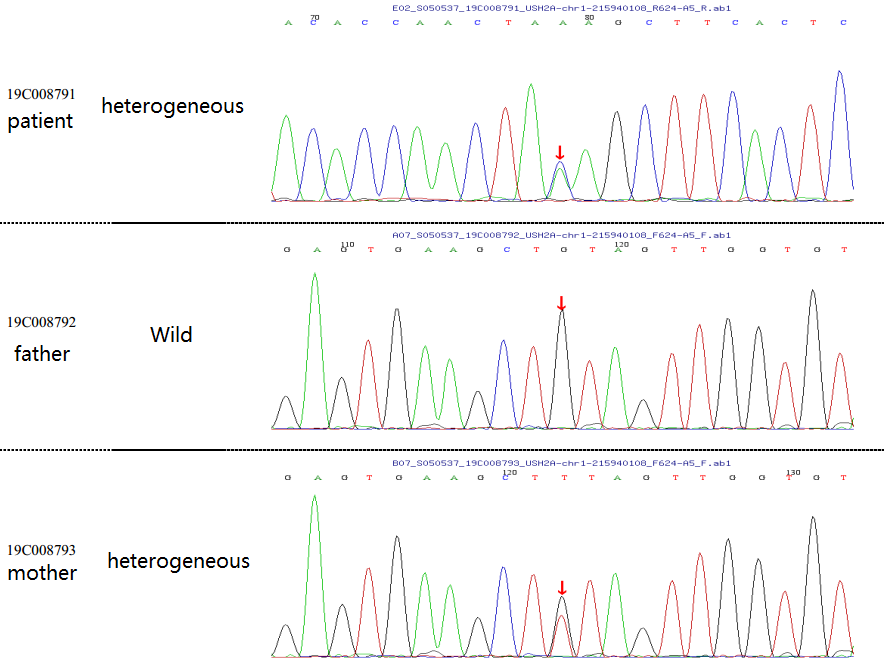


Figure S17. The *USH2A* c.10962C>A mutation in Family 47


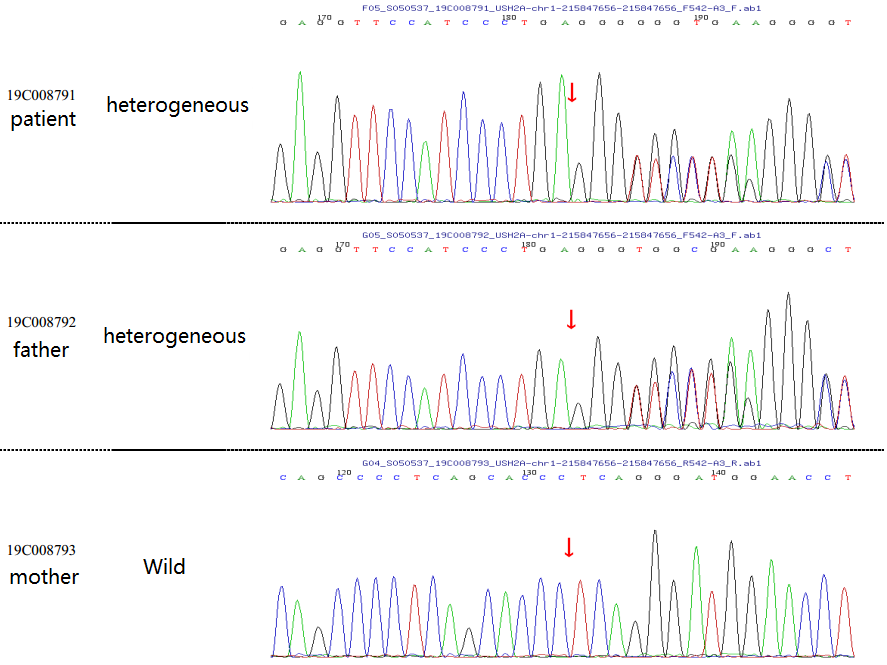


Figure S18. The *USH2A* c.13596dupC mutation in Family 47


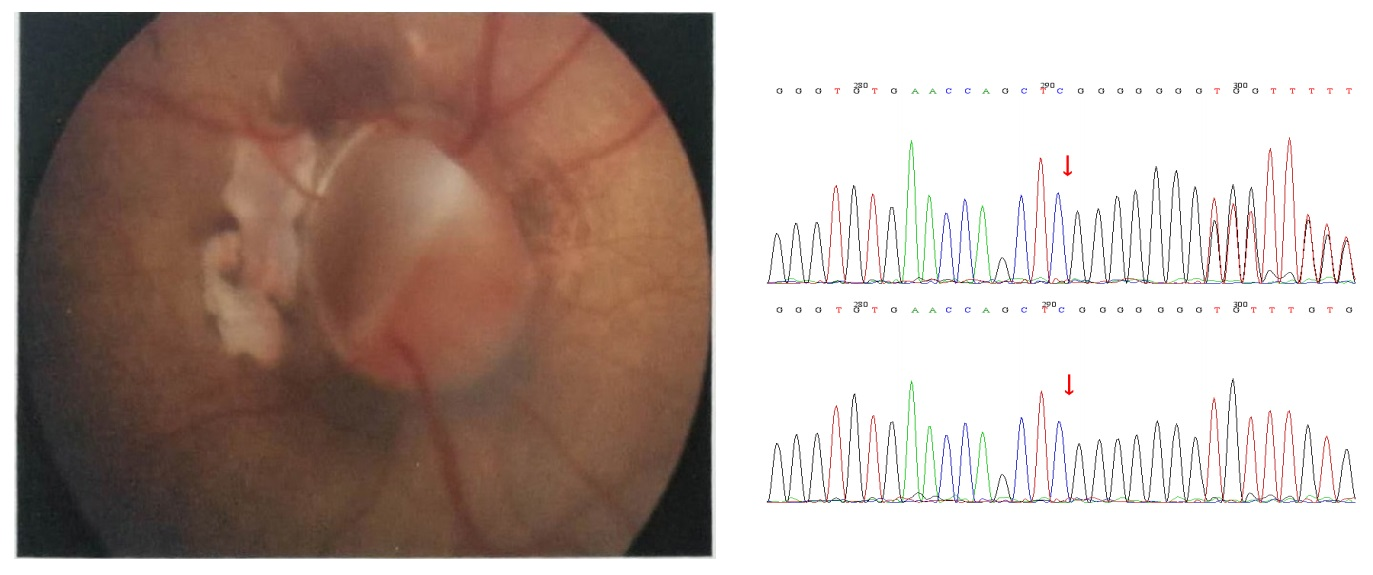


Figure S19. Ophthalmoscopic examination of patient in Family 22 showed the defect of the optic disc and the retinal blood vessels radiation from peripheral optic disc. There is no abnormality in the periphery of the retina.


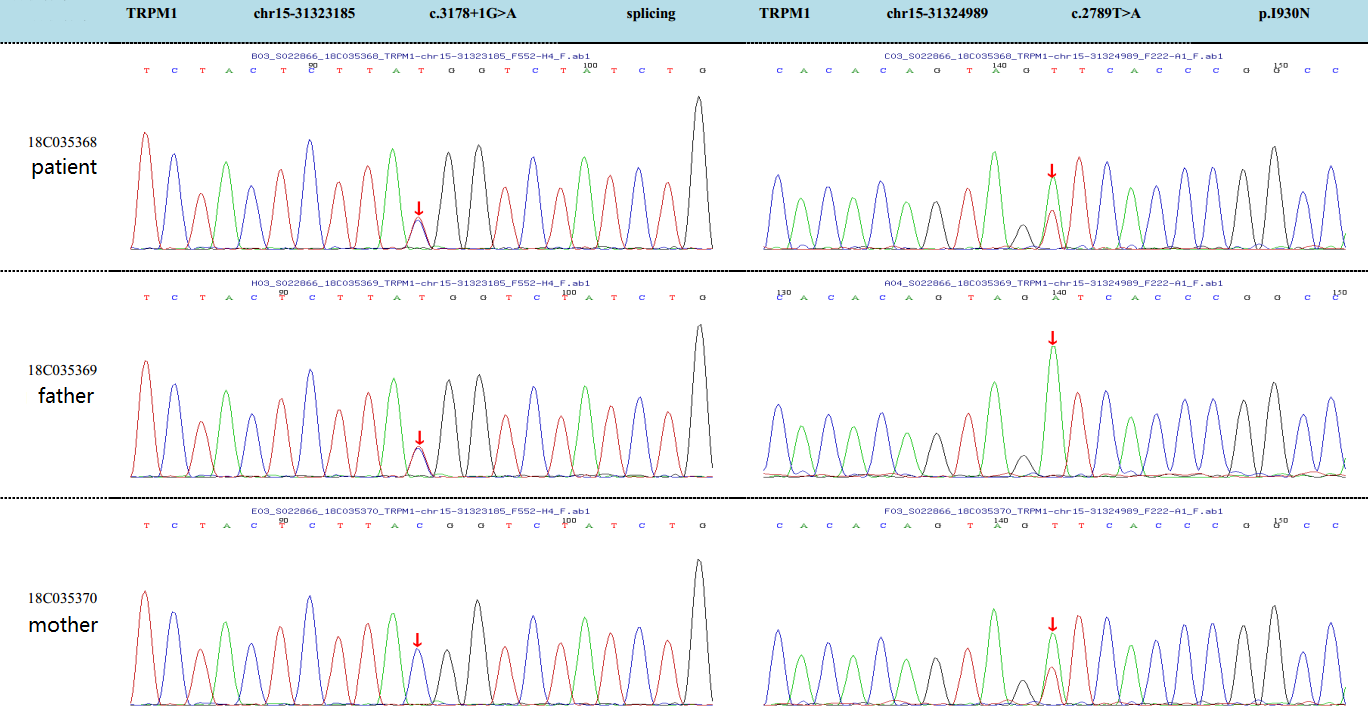


Figure S20. The *TRPM1* c.3178+1G>A and c.2789T>A mutations in Family 20


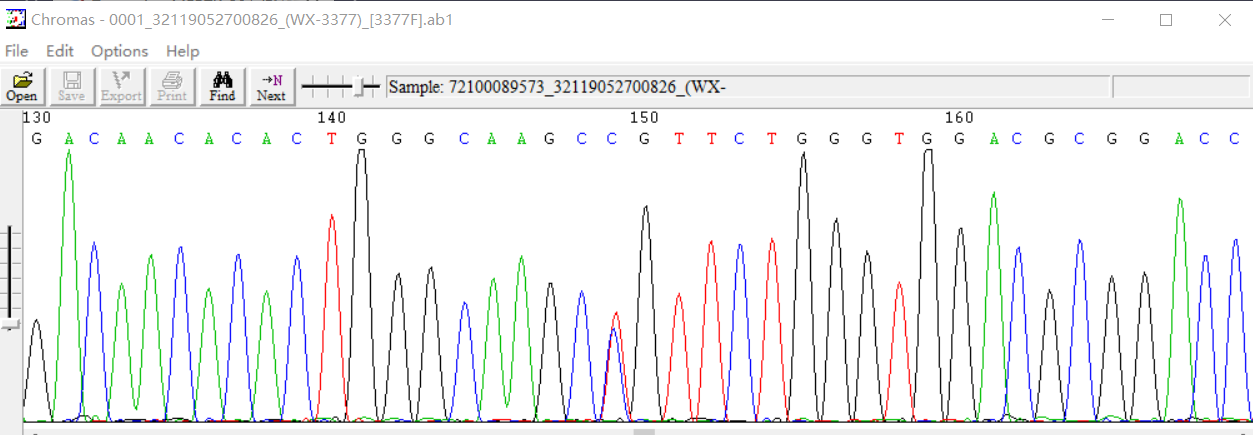


Figure S21. The *LRP5* c.3377T>C mutation in Family 56


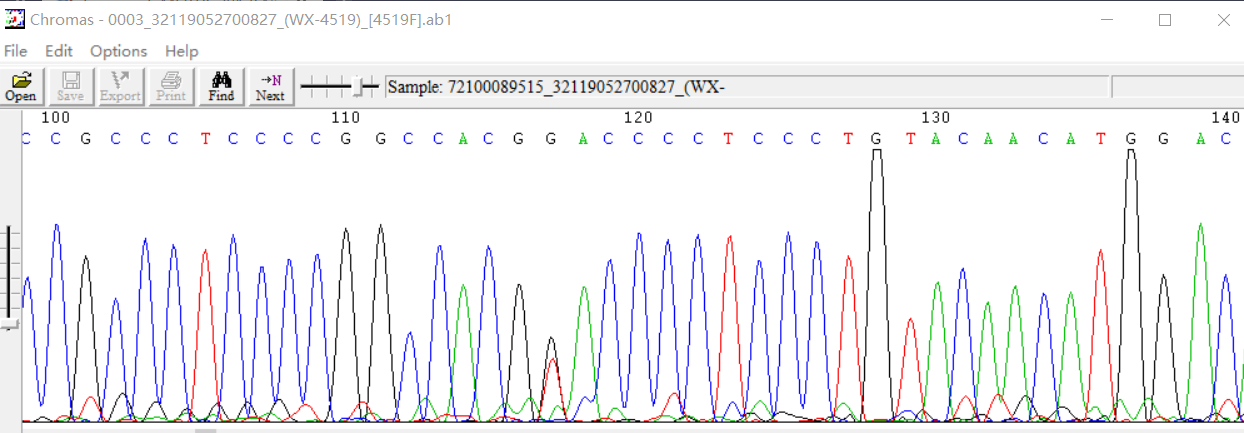


Figure S22. The *LRP5* c.4519G>T mutation in Family 56


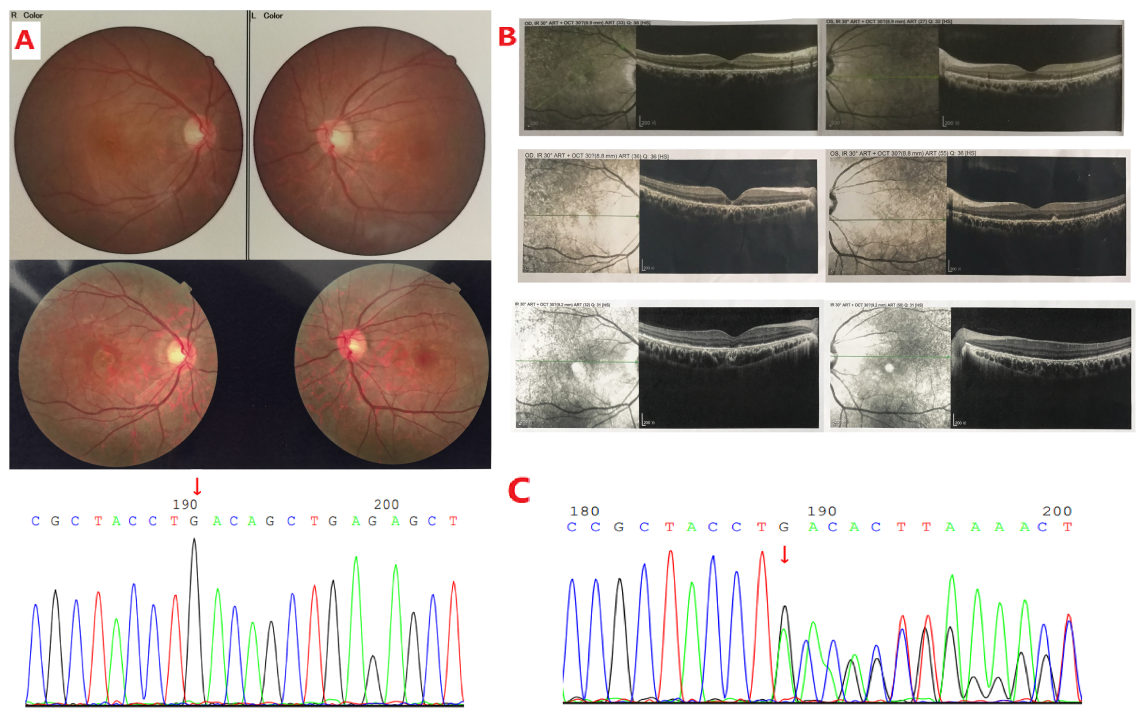


Figure S23. Part A, ophthalmoscopic examination of the patient in Family 5 showed that the RFE layer in macular area of right eye was localized thickening and bulging, and the io/os layer was localized defect. The disc color of binocular fundus was pale. Part B, OCT showed that the signal in the outer layer of the retina was not clear, the pigment epithelium was rough, and the retina exuded between layers. The binocular choroidal signal was enhanced, the right macula was irregular and the IS/OS layer was absent locally, the IS/OS layer of the left macula was blurred. Part C is the Sanger sequencing results of the mutated sites.


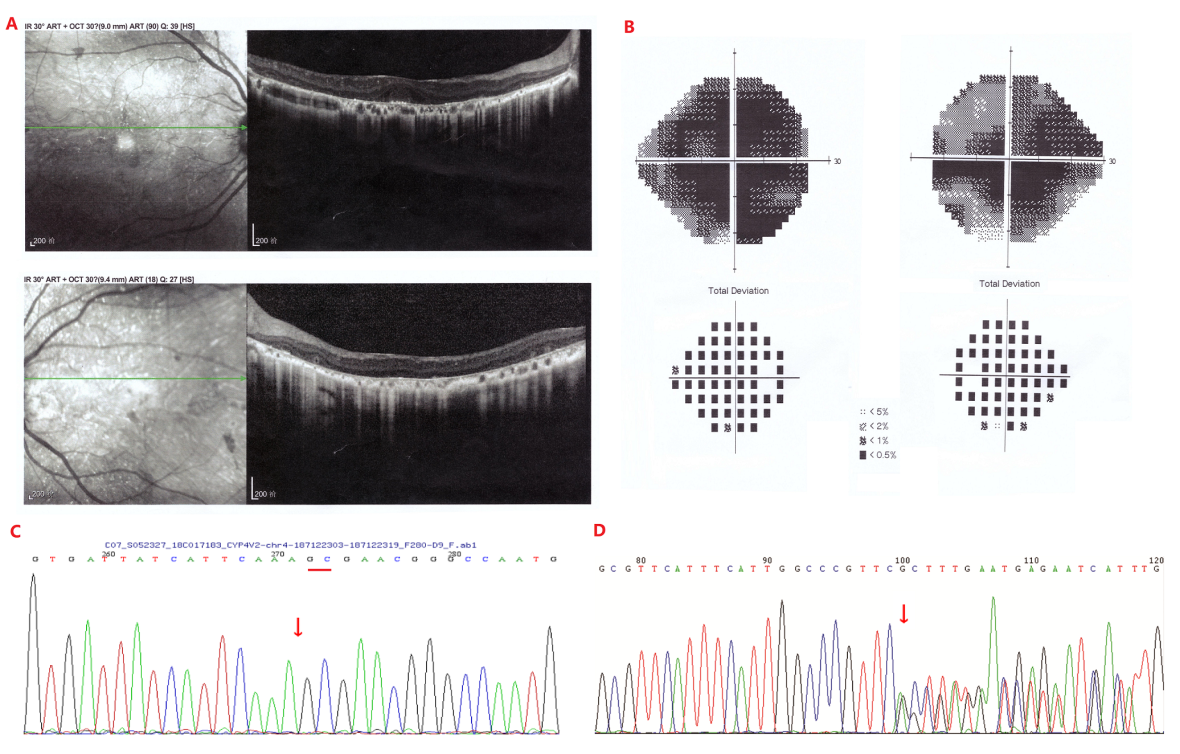


Figure S24. *CYP4V2* mutations and clinical manifestation of the Bietti CCD patient in Family 3. Part A, a large number of crystalline substances can be seen under the neuroepithelial layer of macular area, and a large number of crystalline substances can be seen in the pigmented epithelium and choroidal layer; Part B, centripetal visual field defect; Part C, the patient with homozygous mutation of *CYP4V2* c.(802-8)_810delTCATACAGGTCATCGCTinsGC; Part D, the patient’s daughter with heterozygous *CYP4V2* c.(802-8)_810delTCATACAGGTCATCGCTinsGC.


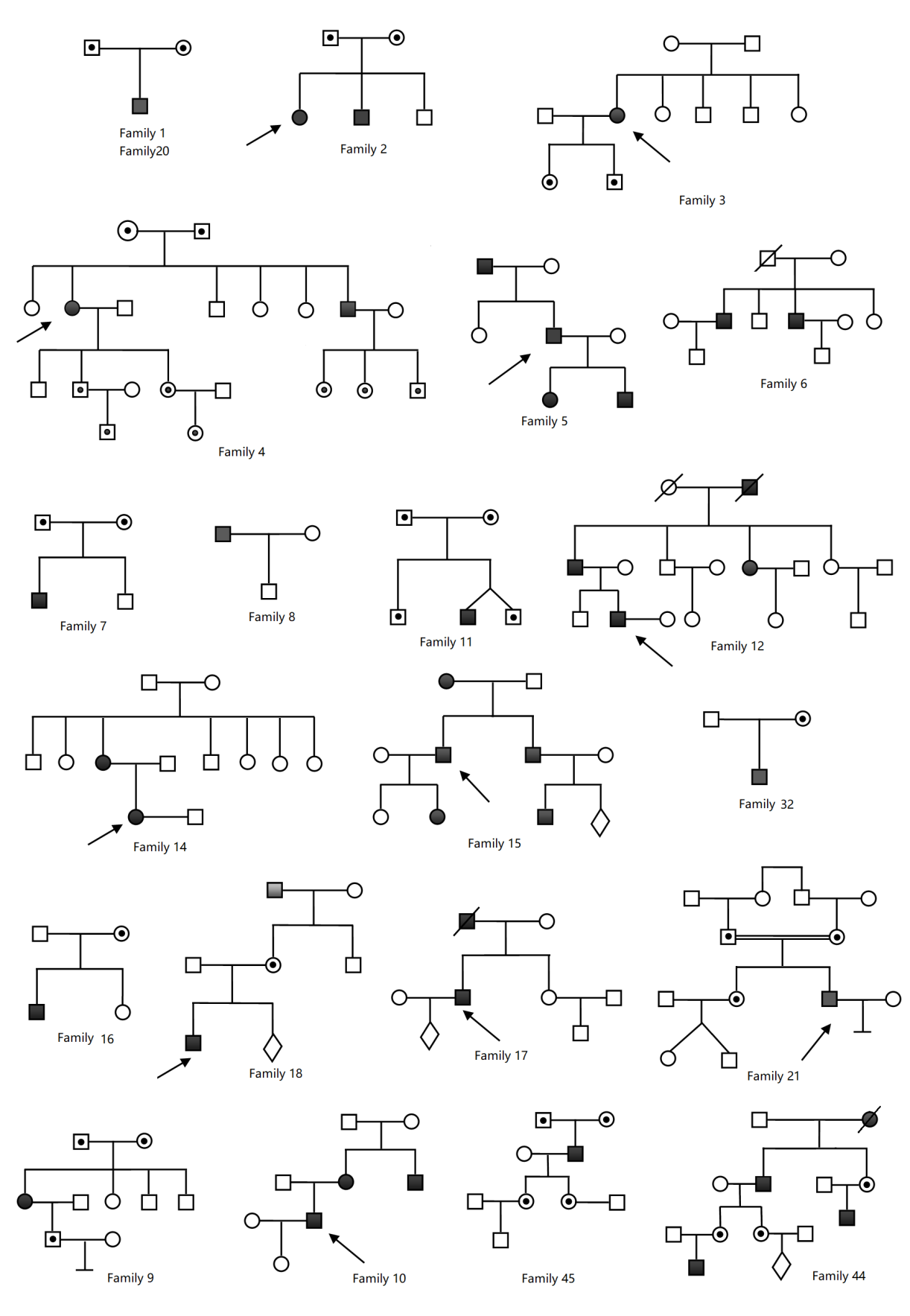


Figure S25. Partial family trees in this study
